# Trimerous magnoliid flowers with a unique set of floral and pollen traits from the Late Cretaceous of Southern Bohemia (Czech Republic)

**DOI:** 10.1111/nph.71310

**Published:** 2026-06-02

**Authors:** Xieting Wu, Maria von Balthazar, Friðgeir Grímsson, Silvia Ulrich, Andrea M. López‐Martínez, Zuzana Heřmanová, Jiří Kvaček, Jürg Schönenberger

**Affiliations:** ^1^ Department of Botany and Biodiversity Research University of Vienna Rennweg 14 1030 Vienna Austria; ^2^ National Museum Václavské náměstí 68 110 00 Praha 1 The Czech Republic

**Keywords:** charcoalified mesofossils, floral morphology, fossil flowers, Late Cretaceous, Piperales, pollen polyads, Southern Bohemia, trimerous flowers

## Abstract

- Floral structure is a key aspect of angiosperm diversity. Recent research revealed that significant floral disparity was already present in the Cretaceous. However, our understanding of early floral diversity remains limited, as it is directly dependent on the fossil record.
- We describe a new, exceptionally well‐preserved flower with *in situ* pollen from the Klikov Formation (late Turonian ‐ Santonian). Our phylogenetic analyses support relationships with the magnoliid order Piperales. Based on a morphospace analysis, we show that the new fossil is among the most morphologically divergent angiosperm flowers.
- *Trimeriantha monopolyada* gen. et sp. nov. represents the first unequivocal evidence of piperalean flowers from the Late Cretaceous and exhibits a unique combination of floral traits, including a single whorl of tepals, three whorls of stamens, anthers with a single pollen polyad per pollen sac and valvate dehiscence, a fusion of androecium and gynoecium, and an extragynoecial compitum. This trait combination distinguishes it from other extant and fossil angiosperms and further expands our understanding of Cretaceous flower diversity.
- Our study adds to the extraordinary morphological diversity known from the Late Cretaceous record of flowers, and we discuss some of the floral traits of *T. monopolyada* with respect to their phylogenetic significance and potential function in pollination biology.

## Graphical abstract

Morphospace position and phylogenetic placements of *Trimeriantha monopolyada*.

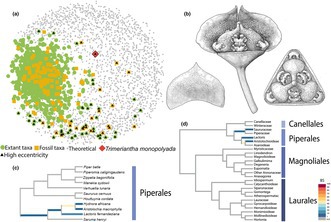

## Introduction

Angiosperms are the most diverse and ecologically dominant plant group on Earth today, and their rapid radiation during the Cretaceous was a major driver for the increase in global biodiversity (Crane *et al*., 1995; Crepet & Niklas, 2009; Benton *et al*., 2022; Dimitrov *et al*., 2023). The question of how angiosperms achieved this rapid radiation has puzzled scientists for decades (Crepet & Niklas, 2009; Sauquet & Magallón, 2018). Numerous hypotheses have been proposed to elucidate this phenomenon, focusing on intrinsic factors, such as the emergence of key traits and epigenetic versatility, or extrinsic factors, such as environmental shifts and the co‐evolution between insects and plants (Crepet & Niklas, 2009; Endress, 2011; Asar *et al*., 2022; Benton *et al*., 2022; Peris & Condamine, 2024). It is undeniable that the flower is one of the most fundamental and significant evolutionary innovations of angiosperms and that it is central to their biology, as it is in the flower where pollination, double fertilization, as well as the production of fruits and seeds take place (Endress, 1994a; Benton *et al*., 2022).

Many extant lineages of angiosperms originated in the Cretaceous (Schönenberger, 2005; Crepet & Niklas, 2009; Friis *et al*., 2011a; Magallón *et al*., 2015; Herendeen *et al*., 2017; Ramírez‐Barahona *et al*., 2020). Fossil flowers from this time not only reveal the extensive diversity of early angiosperms (Crepet *et al*., 2004; Friis *et al*., 2006, 2011a) but also provide insights into the patterns of the early evolutionary history of angiosperms and their flowers. The perianth, for instance, which in Early and mid‐Cretaceous flowers mostly is uniform (i.e. consisting of tepals only), is often differentiated into protective sepals and attractive petals in Late Cretaceous flowers (Friis *et al*., 2010, 2011a). Likewise, while specific nectariferous structures are rare among Early and mid‐Cretaceous flowers, distinct nectary discs are common in Late Cretaceous flowers (Crepet *et al*., 1992; Schönenberger *et al*., 2001; Schönenberger & Friis, 2001; Manchester *et al*., 2018). Such floral innovations play a key role in improving pollination efficiency and thus provide an advantage in complex ecological environments (Dilcher, 2000; Endress, 2011; Benton *et al*., 2022). However, compared to our knowledge about the highly diversified floral morphology in extant angiosperms, our understanding of the morphological and functional diversity of Cretaceous floral structures remains limited.

Recent morphospace analyses through time have shown high floral disparity already in the Early Cretaceous, including traits and trait combinations that are now extinct (López‐Martínez *et al*., 2024). In addition, both the incompleteness of the fossil record and the challenges involved in interpreting isolated specimens indicate that the diversity of early angiosperm floral traits may have been far more extensive than currently recognized (Sauquet & Magallón, 2018; López‐Martínez *et al*., 2024). Therefore, the discovery and study of well‐preserved fossil flowers is essential to refine our understanding of the diversity, morphology, specialization, and ecological roles involved in the early evolution of angiosperms and their floral structures.

In this study, we describe a new flower taxon from the Late Cretaceous of Southern Bohemia (Czech Republic). Multiple well‐preserved fossil specimens provide a detailed picture of this fossil taxon including information on different developmental stages of the flowers. The combination of floral characters is unique among extant and fossil angiosperms but shows clear affinities to the magnoliid order Piperales and in particular to the perianth‐bearing subclade within the order (comprising Aristolochiaceae, Asaraceae, and Lactoridaceae). In contrast to their wide distribution and diversity today, the Cretaceous record of Piperales is scarce and fragmentary, and the order is predominantly represented by fossil leaves, seeds, and fruits (Friis *et al*., 2011b, 2022; Coiffard *et al*., 2014; Meller, 2014; Martinez *et al*., 2015). Here, we present the first convincing evidence of piperalean flowers from the Late Cretaceous. Furthermore, the character combination of this fossil taxon adds significantly to the known morphological diversity of early angiosperms. This discovery offers a fresh perspective on the diversity and complexity of floral innovations during the early evolution of angiosperms.

## Materials and Methods

### Geological setting and fossil assemblage

The sediments containing the numerous charcoalified trimerous fossil flowers described here were collected from the Klikov Formation at the Dobrovodská locality, between Dobrá Voda and České Budějovice (Budweis), Czech Republic. The sediments were exposed during the construction of a new highway from 2021 to 2024, but are not accessible anymore. The Klikov Formation comprises three lithological types with very strong vertical and lateral variations: (1) light gray or yellow conglomerate, coarse‐ to medium‐grain sandstone; (2) red poorly sorted sandy mudstone and sandstone; (3) gray mudstones with organic matter. The lithologies alternate in asymmetrical cycles progressively thinning upward (Slánská, 1976). Based on palynological evidence and the mesofossil record, the age of the Klikov Formation is constrained to the late Turonian‐Santonian (Pacltová, 1981; Knobloch, 1985).

The outcrops of the Klikov Formation produce diverse, abundant, and exceptionally well‐preserved plant mega‐ and mesofossils and palynomorphs, as well as insect eggs and coprolites, many of which yield detailed morphological traits (Knobloch & Mai, 1983, 1986; Heřmanová *et al*., 2021). Until now, the plant fossil assemblage consists of three groups of nonangiosperms, including bryophytes, pteridophytes, and conifers, as well as at least 65 angiosperm species. A notable characteristic of the flora is the high abundance and diversity of plants of the Normapolles complex, an extinct group of eudicots related to core Fagales (Friis *et al*., 2011c), including at least 12 extinct species within five genera based on flowers, young fruits, and pollen (Heřmanová *et al*., 2011, 2016, 2017, 2021, 2023, 2025; Heřmanová & Kvaček, 2012). Another dominant component are capsular fruits and seed fossils, of which several were assigned to the order Ericales (Heřmanová *et al*., 2021).

Paleoclimate estimates based on leaf physiognomy and closest living relatives of the fossil taxa reveal that the Late Cretaceous flora from Southern Bohemia represents a seasonally dry, paratropical‐warm temperate climate with a mean annual temperature of *c*. 15°C (Váchová & Kvaček, 2009). This is also supported by abundant charcoalified and silicified woods (Venclová *et al*., 2023) and reproductive mesofossils (Heřmanová *et al*., 2021).

### Preparation of fossil material

The fossil samples (*Trimeriantha monopolyada* X.‐T.Wu, Balthazar, Grímsson, S.Ulrich, López‐Martínez, Heřmanová, J.Kvaček, Schönenb. sp. nov.) were extracted from the sediment through a process of bulk maceration using sodium bicarbonate, followed by washing with water through sieves with a mesh size of 500 μm and 300 μm. To remove residual mineral material from the specimens, they were then washed with 40% hydrofluoric (HF) and 10% hydrochloric (HCl) acid, rinsed thoroughly in water, and then air‐dried. Subsequently, the material was sorted under a binocular stereomicroscope. Over 200 charcoalified, well‐preserved trimerous flower specimens were found. Ninety specimens were mounted on aluminum stubs with nail polish or adhesive carbon stickers, sputter coated with gold and studied with a scanning electron microscope (SEM; JEOL JSM‐IT300 LV). Ten specimens were scanned with high resolution X‐ray computed tomography (HRXCT; Zeiss/Xradia MicroXCT‐200, 90 keV microfocus X‐ray source (Hamamatsu), 2 k CCD camera, scintillator objective lens units) at the University of Vienna. The HRXCT scanning was carried out with a ×20 optical magnification lens and 1000 projections. For the holotype NM‐F6067, scanning was performed at a voltage of 30 keV with an exposure time of 5 s; for the paratype NM‐F 6117, at 35 keV with an exposure time of 4.5 s; and for NM‐F6118 and NM‐F 6119, both at 35 keV with an exposure time of 4 s. The micro‐CT raw scan data were processed using Avizo 9.4 and Amira 5.5.0 software to obtain 3D models from the image stacks (data from the tomographic scans are made available publicly at https://phaidra.univie.ac.at/o:2343018).

### Preparation of fossil pollen material

For the ultrastructural study of pollen material (preserved *in situ* in fossil flowers), pollen polyads were prepared using the transmission electron microscope (TEM) protocol by Ulrich & Grímsson (2020). Fossil polyads were transferred using a micromanipulator from SEM stubs into embedding molds filled with a mixture of low‐viscosity Agar (LV‐resin) and acetone for infiltration. Following polymerization, ultrathin sections (60–90 nm) were made with a DiATOME Ultra 45 diamond knife on a LEICA Ultramicrotome (LEICA EM UC6, Leica Microsystems GmbH, Wetzlar, Germany) and collected onto formvar film‐coated copper grids. For contrast and to verify the presence of an endexine, sections were stained with 1% aqueous potassium permanganate (KMnO_4_) solution for 5 min (Weber & Ulrich, 2010). The sections were examined using a Zeiss EM900N TEM at 80 kV, with an integrated digital camera (CCD controller), and documented with an Image SP program (ISPViewer64). Panorama scans were made at a magnification of 7.000 for better overviews of the polyads. Picture alignments and picture mergers were made using the Image SP program (ISPViewer64, Unitary Enterprise ‘SYSPROG’, Minsk, Republic of Belarus). The pollen terminology follows Halbritter *et al*. (2018).

### Phylogenetic analyses and visualization of topological uncertainty

The original angiosperm‐wide morphological dataset that we used for the phylogenetic placement of the fossil flowers consists of 30 floral traits recorded for 1201 extant species and 121 fossil species (López‐Martínez *et al*., 2023, 2024) in the PROTEUS database (Sauquet, 2019). These 1201 extant species were sampled to represent the stem and crown nodes of all angiosperm families *sensu* APG IV (2016). The set of floral characters is the same as defined and used by Schönenberger *et al*. (2020), including structural traits describing the sex of flowers (1 character), the perianth (8 characters), the androecium (11 characters), the gynoecium (8 characters), and pollen (2 characters). To these 30 traits of Schönenberger *et al*. (2020), we added three more characters that are important in the fossil flowers described here and scored them for all taxa, for which we could find information: presence or absence of an extragynoecial compitum, partial androecium–gynoecium fusion, and pollen polyads (see Supporting Information Datasets S1, S2). The 33 morphological traits we used here were scored based on an extensive search of data in a wide variety of published literature, including original papers, textbooks, and floras (Sauquet *et al*., 2017; Schönenberger *et al*., 2020; López‐Martínez *et al*., 2023; note that the trait scoring and references for the three additional traits can be found in Dataset S2; the entire data matrix is given as Notes S1).

In addition to the angiosperm‐wide analyses, we tested the phylogenetic position of our fossil flower using the morphological dataset of Doyle & Endress (2024), which consists of 154 morphological characters scored for 66 extant taxa. The taxon sampling of Doyle & Endress (2024) focuses on the ANA grade, magnoliids, and early diverging eudicots, while including only a few taxa of monocots and core eudicots. To this dataset, we added one new character (presence/absence of partial androecium–gynoecium fusion) and one new character state (polyad, added to character 91 of Doyle & Endress, 2024). Presence/absence of an extragynoecial compitum was already present in the dataset of Doyle & Endress (2024; character 115). The morphological dataset, including the scoring data for the fossil flower described here, is provided in Dataset S1 (the entire data matrix is given as Notes S2).

Phylogenetic analyses of both datasets were performed using maximum likelihood (ML) implemented in RAxML v.8.0 (Stamatakis, 2014). In both cases, a backbone tree was used to fully constrain the topology among extant species (RAxML option ‐g). For the López‐Martínez *et al*. (2023) dataset, we employed the maximum clade credibility time tree under the relaxed calibration strategy from Ramírez‐Barahona *et al*. (2020) as a backbone tree (Notes S3). For the Doyle & Endress (2024) matrix, we used the ‘DE MagSister’ tree (Notes S4), in which Magnoliaceae are sister to a clade containing *Degeneria*, *Galbulimima*, *Eupomatia*, and Annonaceae. For both datasets, morphological evolution was estimated with the Markov‐k model (Lewis, 2001) with the ‘lewis’ ascertainment bias correction for the presence of only variable morphological characters and a gamma‐distributed rate variation across characters. For each analysis, we ran a total of 1000 nonparametric bootstrap replicates.

To visualize the uncertainty in the placement of the fossil flower on the angiosperm phylogeny, we generated RoguePlots (Klopfstein & Spasojevic, 2019). These graphs summarize all alternative positions of the fossil on the consensus tree of extant taxa. RoguePlots are based on a set of trees resulting from bootstrapped data matrices and were created using the package rogue.plot (Klopfstein & Spasojevic, 2019) in R (R Core Team, 2023).

### Morphospace analyses

We investigated the position of the fossil flower in the total morphospace of angiosperm flowers using the morphological matrix based on López‐Martínez *et al*. (2024) and three additional characters scored for this study (see previous section). This new matrix consists of 33 discrete characters (identical to those used for phylogenetic assessment) scored for 1201 extant and 121 fossil flowers. To create the morphospace, we followed the methodology described in López‐Martínez *et al*. (2024; the R script is available as ‘Dataset S1’). First, we generated a sample of 2000 theoretically possible floral trait combinations based on the character set, and we then added empirical data from extant and fossil flowers. The distance matrix was estimated by calculating the mean character difference (*D*) for each pair of taxa (for further details on this method, see methods S2 in López‐Martínez *et al*., 2024). The morphospace was visualized using nonmetric multidimensional scaling (nMDS), with the vegan package (Oksanen *et al*., 2020) in R (R Core Team, 2023). Additionally, to determine whether our fossil flower exhibits a distinct floral trait combination among angiosperms, we calculated the eccentricity index (López‐Martínez *et al*., 2024). This index quantifies the divergence of each species from the average morphology by averaging the D for each species from all others in the dataset.

## Results

### Taxonomic description and floral morphology

Order – Piperales *sensu* Helmstetter *et al*., 2025.

Family – incertae sedis.

Genus – *Trimeriantha* X.‐T.Wu, Balthazar, Grímsson, S.Ulrich, López Martínez, Heřmanová, J.Kvaček, Schönenb. gen. nov.

Etymology – The genus name *Trimeriantha* refers to the fact that the flowers (anthos = flower in Greek) are entirely trimerous in the perianth, the androecium, and the gynoecium.

Plant Fossil Names Registry Number: PFN003489.

Generic diagnosis – Flowers pedicellate, actinomorphic, whorled, trimerous, and generally structurally and functionally bisexual. Perianth one‐whorled, undifferentiated, composed of three free, broadly triangular and broadly attached tepals with valvate aestivation. Androecium of three stamen whorls with stamens of the outermost whorl in alternitepalous position and those of the two inner whorls in antetepalous position. Alternitepalous stamens attached to the dorsal, free (superior) portion of the carpels; stamens with filaments, anthers basifixed, dithecate, tetrasporangiate, with flap‐valvate dehiscence. Each pollen sac with a single, spherical pollen polyad composed of 32 pollen grains. Gynoecium tricarpellate‐syncarpous, with a partly inferior ovary. Short styles and stigmas free. Stigmas arranged in close proximity to each other, producing secretion at anthesis and forming an extragynoecial compitum. Ovary trilocular, with axile placentae, at least three ovules per locule.

Species – *Trimeriantha monopolyada* X.‐T.Wu, Balthazar, Grímsson, S.Ulrich, López Martínez, Heřmanová, J.Kvaček, Schönenb. sp. nov.

Etymology – The species name *monopolyada* refers to the presence of a single pollen polyad per pollen sac.

Specific diagnosis – As for the genus with the following additions. Tepals triangular with a glabrous abaxial surface featuring irregularly shaped epidermal cells and interspersed with few stomata, and an adaxial epidermis that is densely papillate in the middle section, transitioning to flat epidermal cells toward the tip and the base. Basifixed anthers slightly longer than filaments; anthers distinctly bilobed. Pollen sacs of a theca well separated from each other, hemispheric in shape and protruding from the thecal tissue. Each pollen sac opening with a flap‐like valve, characterized by papillate epidermal cells. Polyads covered by a continuous‐compact exine layer. Individual pollen grains inaperturate and heteropolar, with regulate to fossulate and perforate exine sculpture. Dorsal carpel surfaces and styles densely covered by glandular papillae.

**Fig. 1:**
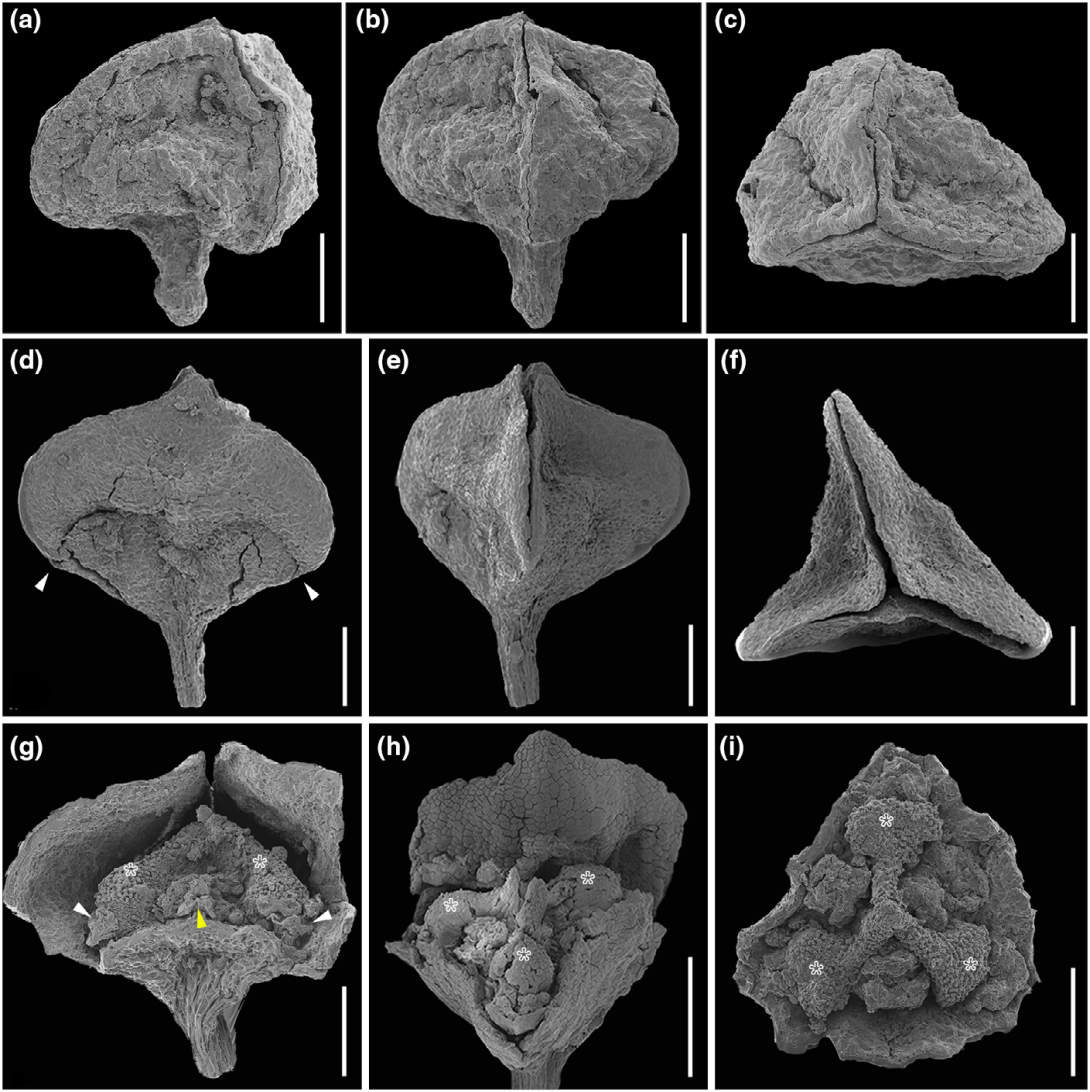
Different specimens of *Trimeriantha monopolyada* shown in different views, partly damaged. (a–c) Intact flower bud with narrow pedicel, NM‐F 6067 (holotype). (a) Lateral view showing a slightly damaged tepal with a broad base, floral base tapering into a narrow pedicel. (b) Lateral view showing the valvate aestivation of the three tepals. (c) Apical view showing the three tepals with reduplicative‐valvate aestivation; note the concave shape of tepals likely caused by fossilization. (d–f) Intact flower bud with narrow pedicel, NM‐F 6069. (d) Lateral view of flower bud showing a well‐preserved tepal, upper margin (arrowheads) of slightly depressed basal area marks area of tepal attachment to floral base. (e) Lateral view showing valvate tepal margins. (f) Apical view showing slightly opened bud. (g) Lateral view of flower bud with one tepal missing, showing two of the three carpels (asterisks) and several stamens in alternitepalous (white arrowheads) and antetepalous (yellow arrowhead) positions, NM‐F 6070. (h) Oblique view of flower bud with two tepals missing, showing three partly damaged carpels (asterisks) and several stamens, NM‐F 6071. (i) Apical view of flower bud with all tepals missing, showing three carpels (asterisks) and nine stamens arranged in three whorls (for details of stamen arrangement, see Fig. 3), NM‐F 6072. Bars, 200 μm.

Holotype – NM‐F 6067 (Fig. 1a–c).

Paratypes – NM‐F 6068–6106, NM‐F 6117–6119 (Figs 1d–i, 2, 3, 4, 5, 6, 7, 8, S1, S2; Dataset S1).

Plant Fossil Names Registry Number: PFN003490.

Repository – The National Museum, Prague, The Czech Republic.

Locality – Dobrovodská, between Dobrá Voda and České Budějovice, the Czech Republic.

Stratigraphy and age‐ Late Turonian‐Santonian, Klikov Formation, Late Cretaceous.

Detailed description and remarks – The material includes numerous (> 200) dispersed, charcoalified, three‐dimensionally preserved flower buds (Fig. 1a–f; Video S1) and anthetic flowers (Figs 1g,i, 3g–i,l, 6c). Some specimens are somewhat compressed or damaged, often lacking tepals or also other floral organs (Fig. 1i). Flowers are described based on bisexual flowers preserved at slightly different developmental stages (Figs 1g–h, 3, 4; developmental stages – pre‐anthetic vs anthetic – can be distinguished based on the size of glandular papillae and the dehiscence of pollen sacs) and one female flower close to anthesis (Fig. 5). Most bisexual flowers are preserved in a pre‐anthetic stage, in which the tepals enclose the remainder of the flower (Fig. 1a–f), while some are preserved at an anthetic or possibly post‐anthetic stage, usually with the perianth partially lacking (Figs 1g–i, 5). The numerous specimens found so far clearly all belong to the same taxon based on the morphological and anatomical details of their individual organs. The flowers are small, 0.43–0.66 mm in diameter and 0.66–0.7 mm in length (without pedicel). Inflorescence type, fruits, seeds, as well as vegetative parts are currently unknown.

**Fig. 2:**
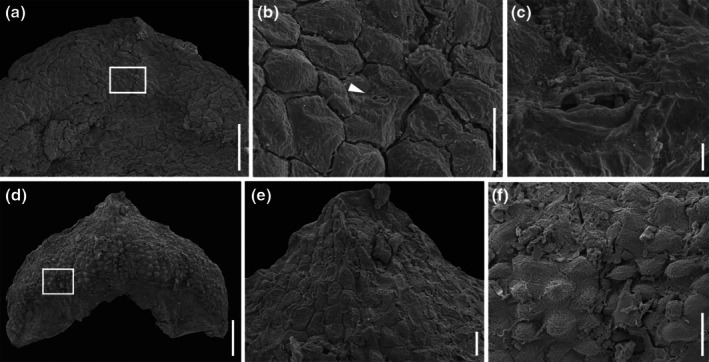
Surface details of tepals of *Trimeriantha monopolyada*. (a–c) Details of abaxial surface of individual tepals, NM‐F 6074. (a) Irregularly shaped epidermal cells on abaxial surface of tepal; white rectangle shows area of the close‐up shown in (b). (b) Close‐up of abaxial tepal surface showing stoma (arrowhead) in the center of the tepal. (c) Close‐up of the stoma shown in (b). (d–f) Details of the adaxial surface of the individual tepal, NM‐F 6075. (d) Adaxial surface of the tepal showing different types of epidermal cells; white rectangle shows area of close‐up shown in (f). (e) Flat epidermal cells close to the tip of the tepal. (f) Distinctly papillate epidermal cells in the middle section of the tepal. Bars: (a, d) 100 μm; (b, e, f) 20 μm; (c) 2 μm.

**Fig. 3:**
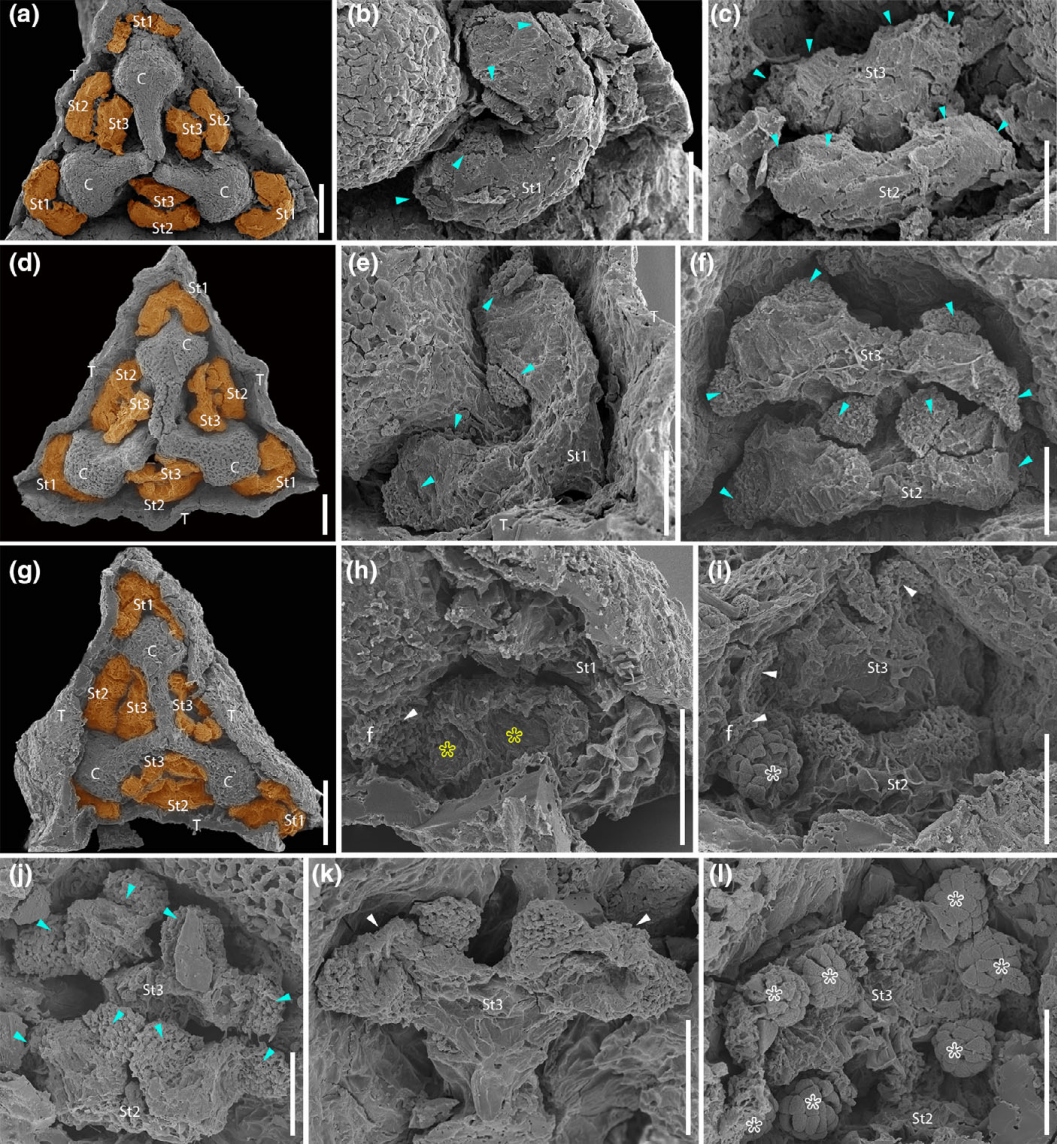
Specimens of *Trimeriantha monopolyada* with all tepals removed or missing, showing androecium structure. (a–c) Pre‐anthetic flower (pre‐anthetic stage indicated by the absence of well‐developed pollen sacs and papillae on the gynoecium surface), NM‐F 6076. (a) Apical view of flower showing bases of three tepals, nine stamens (colored orange), and three carpels. (b) Detail of a stamen (St1) of the outer whorl alternating with the tepals and opposite the carpels; blue arrowheads indicate positions of pollen sacs; note arcuate (curved) shape of anther. (c) Detail of two immature stamens (St2, St3) of the two inner, antetepalous whorls; blue arrowheads indicate approximate position of the immature/damaged pollen sacs. (d–f) A flower close to anthesis (indicated by the presence of distinct papillae on the gynoecium surface and distinct pollen sacs), NM‐F 6077. (d) Apical view of flower with three tepals, nine stamens (colored orange), and three carpels. (e) Detail of a stamen (St1) of the alternitepalous androecium whorl; blue arrowheads indicate position of pollen sacs; note arcuate shape of anther. (f) Detail of two nearly mature, antetepalous stamens (St2, St3); blue arrowheads indicate pollen sacs. (g–i) A flower in anthetic stage (indicated by remnants of stigmatic secretion and dehisced pollen sacs), NM‐F 6078. (g) Apical view of flower showing nine mature stamens (colored orange) arranged in three whorls, two stamens partly covered by tepals. (h) Detail of a stamen of the alternitepalous whorl, showing pollen sacs with flap‐valvate dehiscence; yellow asterisks indicate empty pollen sacs, white arrowhead indicates laterally hinged flap. (i) Detail of two mature, antetepalous stamens (St2, St3); note pollen polyad (asterisk); white arrowheads indicate flaps. (j, k) Close‐ups of Y‐shaped antetepalous stamens in different developmental stages. (j) Two likely pre‐anthetic stamens with pollen sacs still indehisced; note rugose epidermis of protruding pollen sacs; blue arrowheads indicate pollen sacs, NM‐F 6087. (k) Y‐shaped stamen with short, narrow filament and bilobed anther; note sterile tissue between pollen sacs and short thecal protrusions (white arrowheads), NM‐F 6086. (l) Anthetic stamen with dehisced pollen sacs and exposed pollen polyads (asterisks), NM‐F 6079. C, carpel; f, anther flap; St, stamen; T, tepal. Bars: (a, g, j) 100 μm; (b–f, h–i, k–l) 50 μm.

**Fig. 4:**
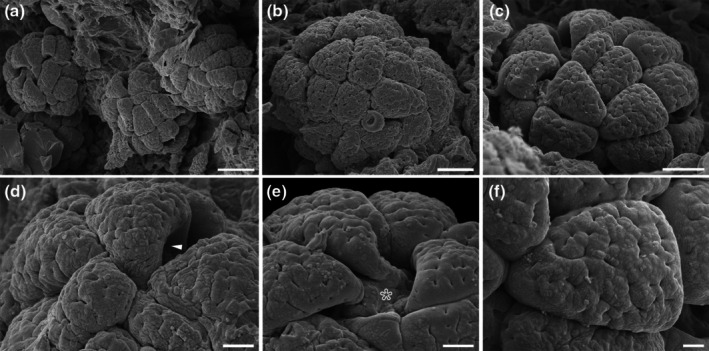
SEM micrographs of pollen polyads of *Trimeriantha monopolyada* preserved *in situ* in a flower specimen, NM‐F 6079. (a) Overview showing four polyads, one partly covered (lower left). (b, c) Close‐ups of polyads showing their spherical shape and the arrangement of individual pollen grains, with rugulate‐fossulate surface sculpture. (d–f) Close‐ups showing the outlines of individual grains and exine surface sculpture. (d) Arrowhead indicates the concave proximal half of a heteropolar pollen grain. (e) Close‐up showing gaps between pollen grains and an outer exine layer (asterisk) partly covering the individual pollen grains (calymmate polyad structure). (f) Close‐up of individual pollen grain with rugulate‐fossulate surface sculpture. Bars: (a) 10 μm; (b, c) 5 μm; (d, e) 2 μm; (f) 1 μm.

**Fig. 5:**
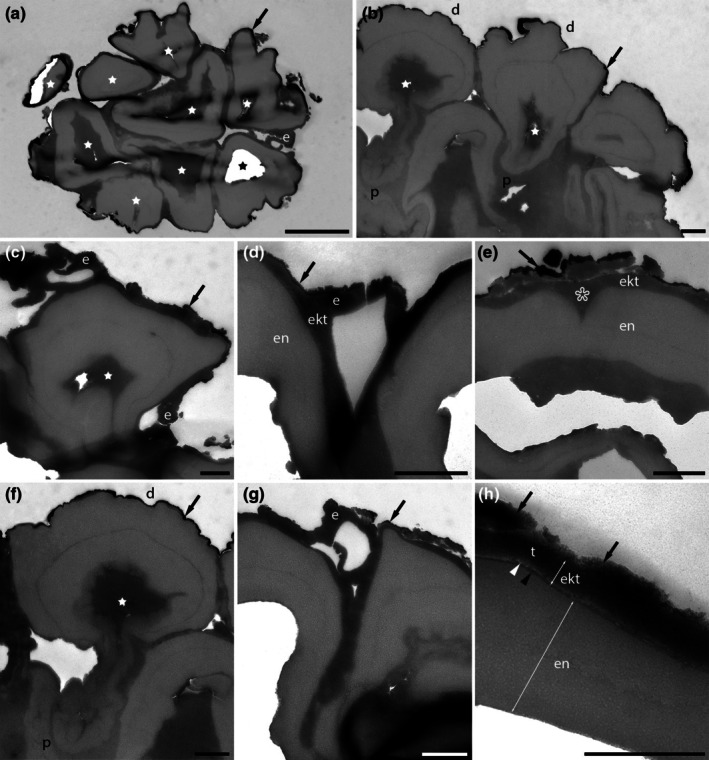
TEM micrographs of pollen polyads of *Trimeriantha monopolyada* showing cross sections of pollen polyads stained with potassium permanganate, NM‐F 6080. (a) Section through polyad showing nine individual grains (marked by stars) in cross section; note compact exine layer extending across the gaps between individual pollen grains and showing that the polyads are calymmate. (b) Close‐up showing arrangement of individual, heteropolar pollen grains (stars) within polyad; note thick pollen wall on distal side decreasing in thickness toward the proximal side. (c, d) Close‐ups showing the exine layer (star indicates individual pollen grain) consisting of a thick endexine and thinner ektexine; the ektexine forms a continuous layer extending across multiple pollen grains; between individual grains, the pollen wall is thicker and composed of ektexine and, in addition, an outer exine envelope. (e) Close‐up of pollen wall showing irregularly distributed crevices (asterisk) extending from the ektexine into the endexine. (f) Close‐up showing heteropolar pollen grain (star) with thick exine on the distal side and thin exine on the proximal side. (g) Close‐up showing ektexine envelope covering polyad. (h) Close‐up showing exine of pollen grains located on the periphery of the polyad composed of a thin ektexine (white double arrow) and a thick endexine (long, white double arrow); ektexine composed of a very thin foot layer (black arrowhead), a thin and inconspicuous granular infratectum (white arrowhead), and an eutectate tectum. Note that the sample was previously studied in SEM; therefore, it is covered by a thin but electron‐dense gold layer (black arrows). d, distal; ekt, ektexine; en, endexine; p, proximal; t, tectum. Bars: (a) 5 μm; (b–h) 1 μm.

**Fig. 6:**
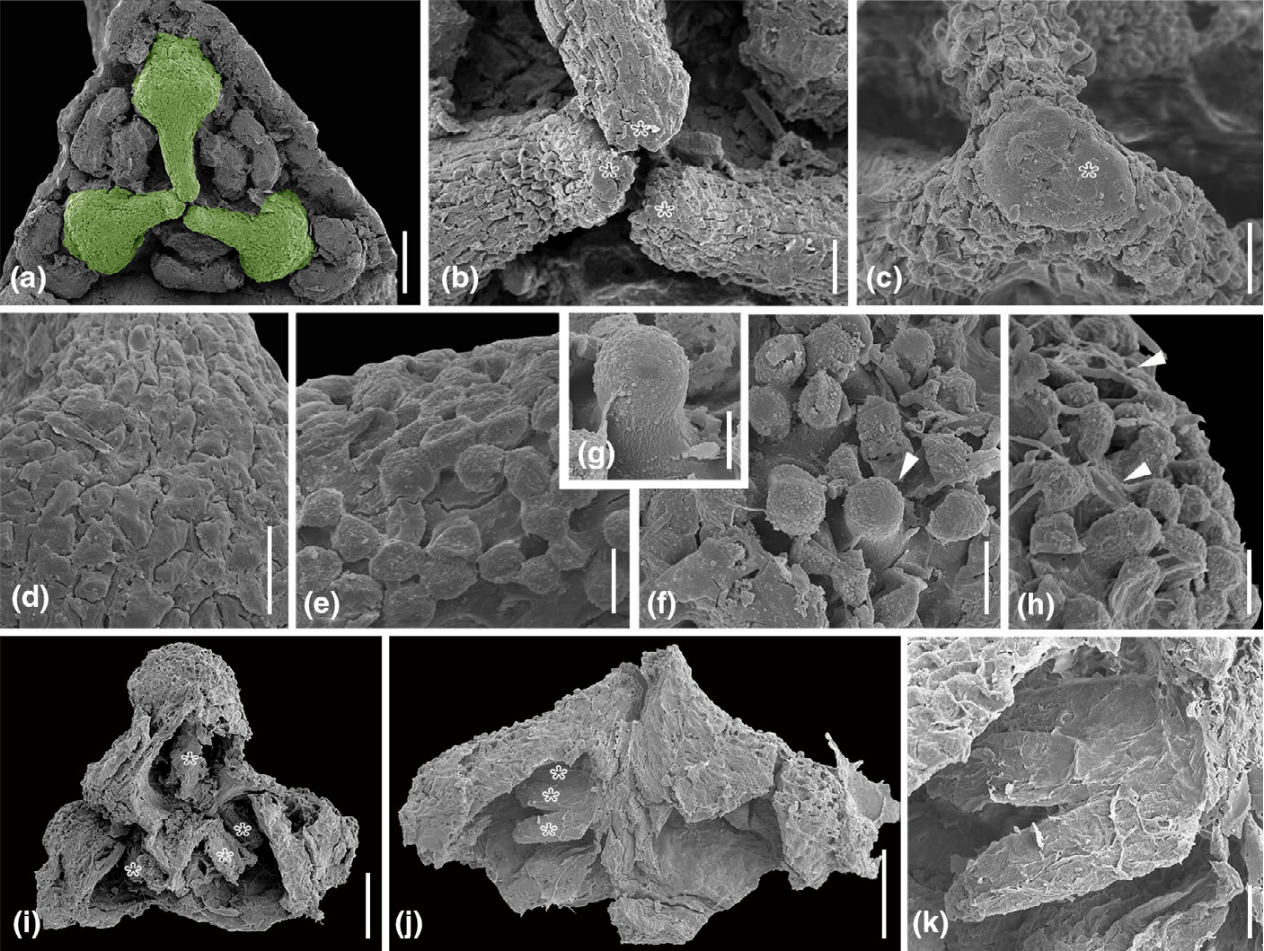
Details of the gynoecium structure of *Trimeriantha monopolyada*. (a, b) Apical view of pre‐anthetic flower, tepals not preserved, NM‐F 6076. (a) Carpels colored green; note the dorsal bulging of the superior part of carpels and styles directed toward the center of the flower. (b) Close‐up of styles and premature stigmas; note that tips of styles and stigmas (asterisks) are free from each other. (c) Apical view of style and stigmas of a flower preserved in an anthetic stage; note remnants of a drop of secretion forming an extragynoecial compitum (asterisk) covering all three stigmas, NM‐F 6081. (d–h) Surface details of carpels. (d) Irregularly shaped, nonpapillate epidermal cells on carpel surface at a pre‐anthetic stage of development, NM‐F 6076. (e) Shortly papillate epidermis cells on carpel surface at a stage close to anthesis, NM‐F 6077. (f) Papillate, glandular epidermis cells on carpel surface at the anthetic stage; arrowhead indicates individual papillate epidermis cell shown in (g), NM‐F 6070. (g) Close‐up of the individual papillate epidermal cell. (h) Papillate, glandular epidermis cells on carpel surface at the anthetic stage; arrowheads indicate potential remnants of secretion (and possibly also fungal hyphae), NM‐F 6082. (i) Bottom view of dissected ovary showing ovules (asterisk) borne in a row on axile placenta, NM‐F 6083. (j) Dissected ovary showing two damaged carpels with three ovules (asterisks) per locule, NM‐F 6084. (k) Close‐up of the ovules in (i). Bars: (a, i, j) 100 μm; (b, c, k) 20 μm; (e, f, h) 10 μm; (g) 5 μm.

**Fig. 7:**
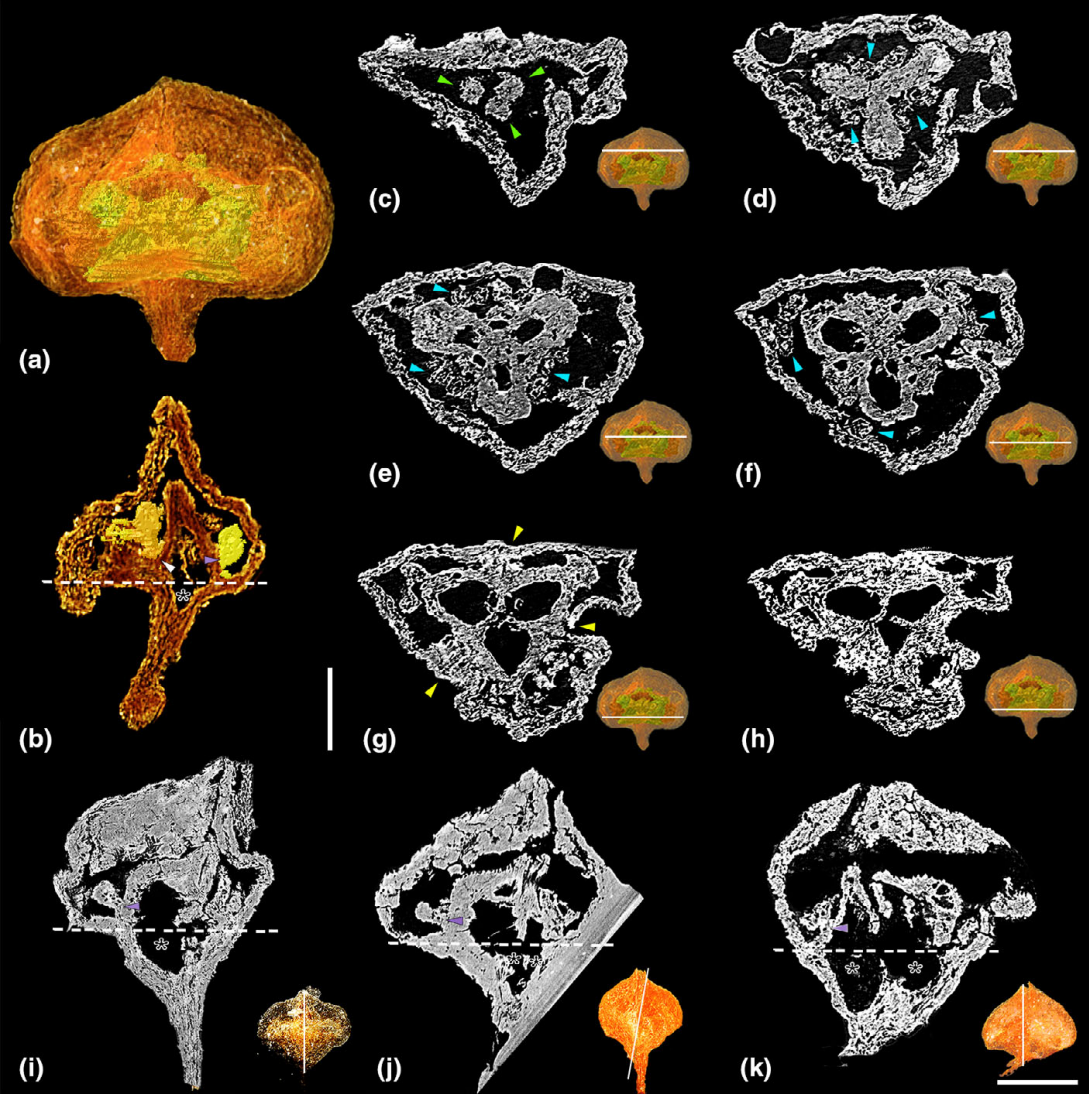
Tomographic scan of bisexual flowers of *Trimeriantha monopolyada*. (a–h) NM‐F 6067 (holotype). (a) Volume rendering of bud, composed of outer tepals and pedicel (colored orange), inner stamens (colored yellow and brown), and carpels (colored green). (b) Longitudinal section through flower bud, showing partly inferior ovary (dashed line indicates approximate level of tepal insertion, asterisk indicates inferior part of ovary locule; note that the specimen is slightly compressed along the basal‐apical axis); the alternitepalous stamen (in yellow) is fused to the dorsal side of the superior part of a carpel (purple arrowhead) and the two antetepalous stamens are inserted on the floral base between the flanks of two carpels (white arrowhead). (c–h) Series of transverse sections, from tip (c) to base (h) of bud. (c) Distal part of flower bud with tepals and tips of free styles (green arrowheads). (d) Level of stamens (blue arrowheads) of inner antetepalous stamen whorl. (e) Level of stamens of outer antetepalous stamen whorl (blue arrowheads). (f) Level of stamens of alternitepalous stamen whorl (blue arrowheads). (g) Level at which tepals, antetepalous stamens, and carpels are united in the radii between the carpels (yellow arrowheads). (h) Level of floral base with inferior part of ovary. (i–k) Longitudinal sections through the median plane of one of the carpels in three different flower specimens, showing the partially inferior ovary (asterisks indicate inferior parts of ovary locules; dashed lines indicate level of tepal insertion) and gynostemium (purple arrowhead indicates point of insertion of stamen on the dorsal side of the carpel), NM‐F6117, NM‐F6118, and NM‐F6119. Bars, 200 μm.

**Fig. 8:**
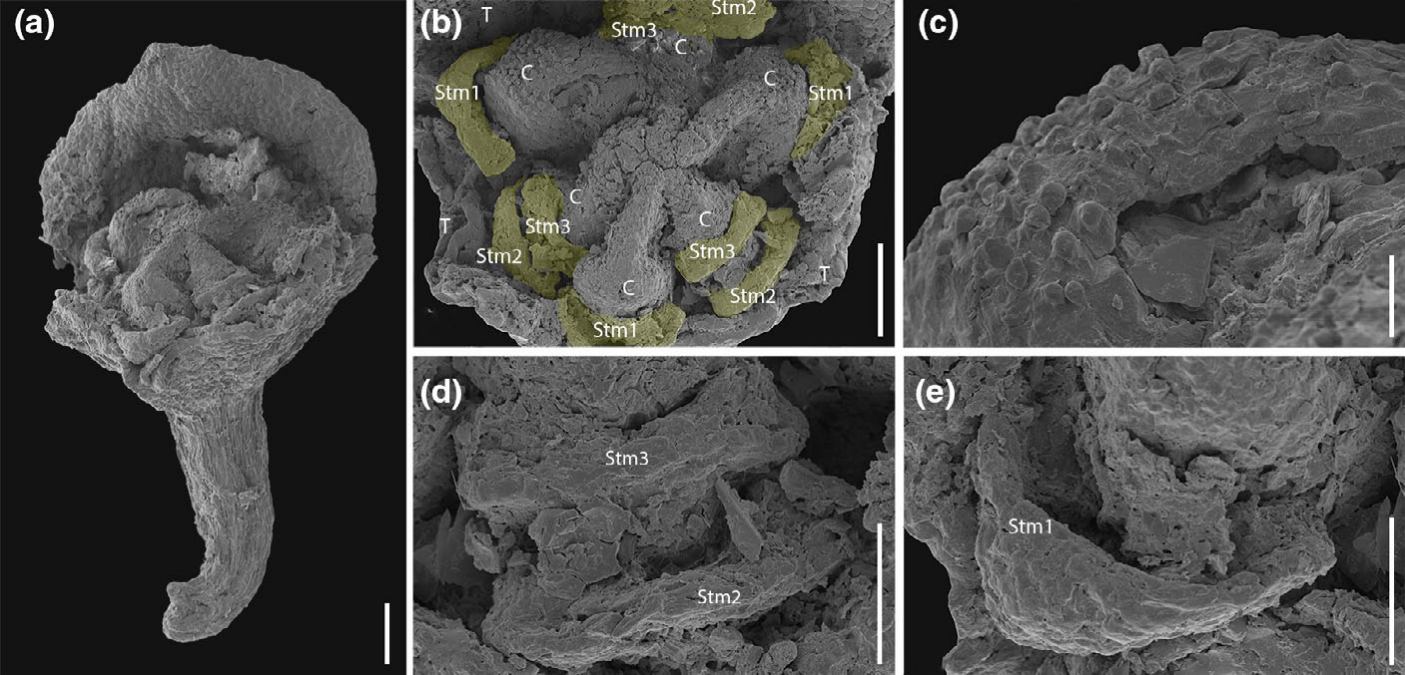
Functionally female flower of *Trimeriantha monopolyada* with three whorls of staminodes and six carpels, NM‐F 6085. (a) Lateral view of the whole specimen with a long pedicel, two tepals broken off. (b) Apical view showing nine staminodes (arranged in three whorls; Stm, colored yellow) and six carpels (c). (c) Detail of the dorsal surface of one of the carpels covered with glandular‐papillate epidermis cells. (d) Detail of two staminodes (Stm2, Stm3) in antetepalous position; note that no pollen sacs are differentiated on the staminodes. (e) Detail of a staminode (Stm) in alternitepalous position; note that no pollen sacs are differentiated on the staminode. C, carpel; P, petal; T, tepal; Stm, staminode. Bars: (a, b) 100 μm; (c, e) 50 μm; (d) 20 μm.

#### Bisexual flowers

##### Perianth

The perianth is undifferentiated and comprises a single whorl of three tepals (Figs 1a–f, 2). Tepals are free, equal in size and shape, with distinctly valvate aestivation; the margins of neighboring tepals are bent outward, the aestivation thus being reduplicate‐valvate (Fig. 1c,f). Individual tepals are broadly equilaterally triangular in outline, 0.32–0.5 mm long, 0.85–1 mm wide (Figs 1a,d, 2a,d), and *c*. 20 μm thick, consisting of several cell layers (Fig. 1b,c,e,f). The tepal apex is more or less acute (Figs 1a–f, 2a,d,e). The tepal base is truncate, slightly narrower than the widest part of the tepal (Figs 1a,d, 2a,d), and broadly attached to the floral base, which encloses the partially inferior ovary (Fig. 1a,d). The abaxial surface of the tepals is glabrous, consisting of approximately isodiametric, but irregularly shaped epidermal cells and occasional stomata (Fig. 2a–c). On the adaxial surface, the tepals display densely spaced papillate cells in the middle section (Fig. 2d,f), while epidermal cells are flat toward the tip and base (Fig. 2e).

##### Androecium

**Fig. 9:**
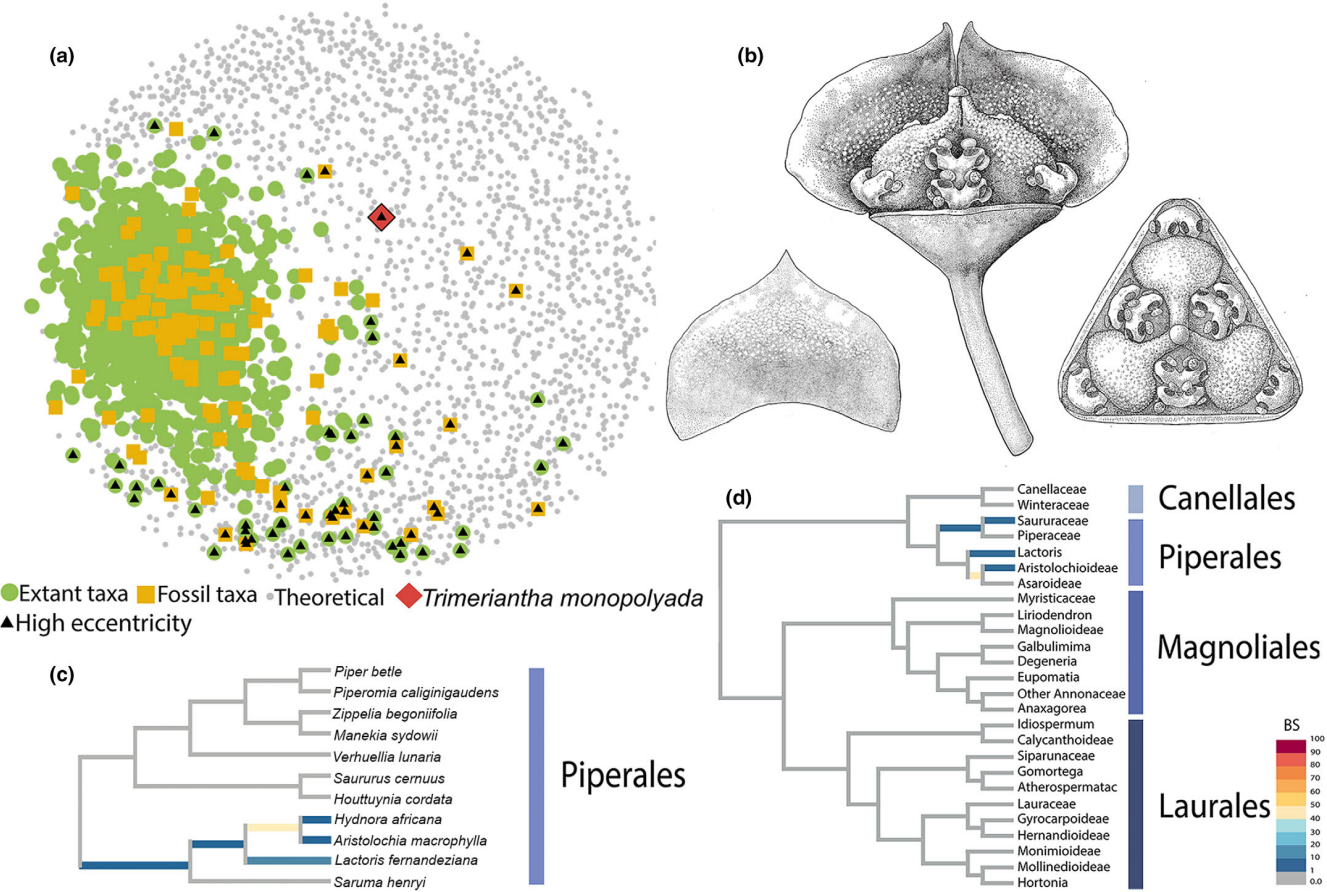
Visualizations of morphospace and phylogenetic analyses of *Trimeriantha monopolyada*. (a) Position of *T. monopolyada* (red diamond) and the 5% of most eccentric species (black triangles) in the floral morphospace of angiosperms (data from López‐Martínez *et al*., 2024). (b) Reconstructed illustrations of *T. monopolyada*, displaying from left to right: an individual tepal, a lateral view of the whole flower, and a top‐down (aerial) view; drawing by Julia Asenbaum. (c, d) Partial RoguePlots (for full trees, see Supporting Information Dataset S1; Figs S1a–c) showing alternative phylogenetic positions of *T. monopolyada* and their associated uncertainty; note that the branches are colored according to the bootstrap values (BS values in color legend) associated with the attachment of the fossil to the branch; colors in the legend at right range from blue to red and indicate increasing bootstrap support values; higher values indicate greater support for attachment of the fossil to a given branch. (c) Partial RoguePlot based on the maximum likelihood analysis using the angiosperm‐wide matrix based on López‐Martínez *et al*. (2023). (d) Partial RoguePlot estimated with the maximum likelihood analysis of the morphological matrix focused on noneudicot angiosperms based on Doyle & Endress (2024).

The androecium comprises three whorls of three stamens each (Fig. 3a,d,g). The outermost whorl of stamens alternates with the tepals (Fig. 3a,b,d,e,g,h), while the stamens of the two inner whorls are both positioned in the radii of the tepals (Fig. 3a,c,d,f,g,i). The anthers of all stamens are dithecal, tetrasporangiate, and basifixed (Fig. 3j–l). The short filaments of the alternitepalous stamens are fused to the base of the superior part of the carpels (Fig. 7b,i–k), and the anthers are *c*. 150 μm broad (Fig. 3b,e). The filament of the antetepalous stamens is *c*. 40 μm long, and the anther *c*. 160 μm broad (Fig. 3j–k). Individual anthers are distinctly bilobed and curved (arcuate, *sensu* Hufford, 1996), and pollen sacs are located laterally on the broad thecae (Fig. 3a,b,d,e,g,h). The arcuate shape of the anthers and the lateral position of the pollen sacs on the thecae result in slight differences in the direction of anther dehiscence among the pollen sacs of an individual stamen (see also below). Pollen sacs are globose and protruding (Fig. 3b,c,e,f,h,k). The two pollen sacs of a theca are clearly separated from each other, as the sterile tissue between the two pollen sacs of a theca is extensive (Fig. 3b,c,e,f), and in the antetepalous stamens even extends into a short protrusion (Fig. 3k). Each pollen sac opens with its own, laterally hinged, flap‐like valve (Fig. 3b,c,e,f,h,i), which is characterized by distinctly papillate epidermal cells (Fig 3e,f,h,i). These papillate cells appear collapsed in dehisced stamens (Fig. 3h–k). Individual pollen sacs each contain a single, spherical pollen polyad (Figs 3i,l, 4a–c). Thus, each stamen produces only four pollen polyads. The anthers of the alternitepalous stamens differ slightly in shape and orientation from those in the antetepalous position. The anthers of the former are more strongly arcuate with the two thecae oriented horizontally (Fig. 3b,e), whereas the latter are less pronouncedly arcuate and the thecae are in a more upright position, giving them a distinctly Y‐shaped appearance when observed from the side (Fig. 3j,k). The differences in thecal shape and orientation between the stamen whorls result in differences in the direction of anther dehiscence, with pollen release in the alternitepalous stamens directed more toward the floral center (apical‐introrse, Figs 3b,e, 9b) and that of the antetepalous stamens toward the floral periphery (extrorse, Figs 3l, 9b).

##### Pollen

Pollen polyads are 23.3–31.5 μm in diameter (longest), 24.75 μm on average (15 polyads measured on SEM images). Polyads are each composed of 32 pollen grains and are calymmate, that is, they are covered by a thin, continuous layer of ektexine that varies in thickness (0.2–0.32 μm measured on TEM images) (Figs 4d–f, 5a). Individual pollen grains are inaperturate and heteropolar, circular or triangular to hexagonal in outline in distal polar view (Figs 4d–f, 5a,b). They are 6.3–10.7 μm in diameter (longest), 7.45 μm on average (43 grains measured, SEM). Pollen grains appear relatively loosely packed with gaps between individual grains (Figs 4c–e, 5a). The exine sculpture is rugulate to fossulate and perforate, granulate (Fig. 4e,f), and composed of a thin ektexine and a thick endexine (Fig. 5c–e,h) with irregularly distributed crevices extending from the ektexine into the endexine (Fig. 5c,d) (corresponding to fossulae observed with SEM, Fig. 4e,f). The exine is thickest on the distal side of the grains and decreases in thickness toward the proximal side (Fig. 4c,d). The ektexine is 0.25–0.44 μm thick in the distal half of pollen grains and 0.05–0.1 μm thick in the proximal half. The endexine is 0.86–1.22 μm thick in the distal half and 0.47–0.52 μm thick in the proximal half (Fig. 4d,e,h). The ektexine is composed of a thin foot layer, 0.013 μm thick, and a thin and inconspicuous granular infratectum, 0.03–0.04 μm thick, and an eutectate tectum (Fig. 4h). The tectum varies in thickness from 0.22 to 0.27 μm in the distal half and from 0.05 to 0.1 μm thick in the proximal half of the grains (measured on TEM images).

##### Gynoecium

The gynoecium consists of three carpels in alternitepalous position (Fig. 6a) that are united at the level of the ovary but have free styles (Figs. 6a,i, 7). The ovary is trilocular and partly inferior, with approximately one third of the ovary inferior and two thirds superior and free from the floral base (Fig. 7b,h–k). In the superior part of the ovary, the carpels are dorsally bulging and extending toward the periphery of the flower (Figs 6a, 7b), whereas the three short, free styles are directed toward the floral center. The three small stigmas at the tips of the styles are *c*. 35 μm in diameter and are arranged close to each other in the center of the flower (Figs 6a, 7c). In specimens that were preserved at anthesis, stigmas show remnants of secretion covering all three stigmas and thereby forming an extragynoecial compitum (36–51 μm in diameter) (Fig. 6c). Each ovary locule contains at least three ovules (Fig. 6h–j). The ovules are *c*. 100 μm long and 30 μm wide and are borne in a row along an axile placenta in the superior part of the ovary (Fig. 6h–j). Other details of the ovules (e.g. curvature or integument number) cannot be established based on the available material.

In the superior part of the gynoecium, the carpels' surface is densely covered by unicellular, slightly capitate, likely glandular trichomes (Fig. 6d–g, note remnants of secretion in Fig. 6h). These trichomes apparently develop only relatively late during floral development as the carpel wall is more or less smooth and composed of irregular epidermal cells in younger, pre‐anthetic flowers (Fig. 6d).

#### Functionally female flower

One of the numerous specimens represents a functionally female flower, similar in size and appearance to the bisexual flowers (Fig. 8a). The flower is composed of three tepals, nine staminodes, and six carpels (Fig. 8b). The one preserved tepal of the specimen is equivalent in shape, size, and surface details to those of the bisexual flowers described above (Fig. 8a). The androecium comprises three whorls of three scale‐like staminodes each, as in the bisexual flowers with one alternitepalous and two antetepalous whorls (Fig. 8a,b). Similar to the bisexual flowers (Fig. 2j–l), the alternitepalous staminodes are more curved than the antetepalous ones (Fig. 8a–c,e). They appear to be staminodes as no pollen sacs are differentiated. The six carpels appear partly deformed, likely through compression during fossilization. The three carpels that alternate with the tepals are larger than the other three (Fig. 8b). The fully developed glandular trichomes on the dorsal carpel surface (Fig. 8d) indicate that this female flower was preserved close to or during anthesis.

#### Phylogenetic analyses and morphospace occupation

Our phylogenetic analyses, based on two independent morphological matrices, both support the position of *T. monopolyada* within the order Piperales (Figs 9c,d, S3; Dataset S1). The ML analysis based on the López‐Martínez *et al*. (2023) dataset supports different positions of *T. monopolyada* within Piperales, with the highest support for the stem branch leading to Aristolochiaceae plus Hydnoraceae (40–50 BS; Figs 9c, S3a,b; Dataset S1). Our analysis based on the Doyle & Endress (2024) matrix shows the highest support for the placement of *T. monopolyada* within Piperales (40–50 BS; Fig. 9d). However, it also recovered some other positions with slightly weaker support within the monocot clade, particularly on the branch leading to *Acorus* (30–40 BS; Dataset S1; Fig. S3c), and several early diverging eudicots (Dataset S1; Fig. S3c).

Our morphospace analysis places *T. monopolyada* in the middle portion of the theoretical morphospace of angiosperms (Fig. 9a), showing minimal overlap with the area of highest occupancy of fossil and extant flowers. Our estimation of the eccentricity index indicates that *T. monopolyada* is among the 5% of angiosperm species with the most divergent structural floral morphologies (black triangles; Fig. 9a). The list of the most eccentric species is provided in Dataset S1; Table S1.

## Discussion

A recent morphospace study estimating the morphological diversity (=disparity) of flowers through time has shown that angiosperms reached high levels of floral disparity already during early stages of their evolutionary history in the Early Cretaceous (López‐Martínez *et al*., 2024). With its unique character combination and special floral features, *T. monopolyada* further adds to the known disparity of Late Cretaceous flowers. Our morphospace analysis (Fig. 9a; Dataset S1; Table S1) shows that *T. monopolyada* is among the 5% of the most eccentric (i.e. most divergent from the average morphology) species of all extant and fossil angiosperms included in the analysis of López‐Martínez *et al*. (2024). The unusual combination of floral characters includes a single whorl of perianth organs, three whorls of stamens, flap‐valvate anther dehiscence, pollen likely dispersed as polyads, the presence of partial androecium–gynoecium fusion, and an extragynoecial compitum formed by secretion. These distinctive and specialized traits strongly support the morphological and most likely also functional uniqueness (see subsequently) of the *T. monopolyada* flower.

### Systematic affinity and floral structure

In spite of the unique character combination of *T*. *monopolyada*, our phylogenetic analyses – one at the level of angiosperms as a whole (Figs 9c, S3a,b; Dataset S1) and one with a focus on noneudicot lineages (Figs 9d, S3c; Dataset S1) – both support a relationship with the magnoliid order Piperales. The order comprises *c*. 4200 extant species in six families (Saururaceae, Piperaceae, Lactoridaceae, Hydnoraceae, Asaraceae, and Aristolochiaceae; Nickrent, 2020; Jost *et al*., 2021; Helmstetter *et al*., 2025). Piperales display a high diversity at the level of floral architecture (*sensu* Endress, 1994a; Dataset S1; Fig. S4a), comprising both some of the largest and most complex flowers (e.g. in the genus *Aristolochia*, Davis *et al*., 2008), besides some of the smallest and simplest flowers (e.g. in the genus *Peperomia*, Tucker, 1980) among extant angiosperms.

Despite their architectural diversity, most extant Piperales share a series of floral characters at the level of floral organization (*sensu* Endress, 1994a) not only with each other but also with *T. monopolyada*: flowers are generally bisexual, actinomorphic (except *Aristolochia* L.), trimerous, and have a whorled phyllotaxis. However, based on angiosperm‐wide ancestral state reconstructions, the combination of these four traits has been identified as most likely ancestral for angiosperms (Sauquet *et al*., 2017) and, therefore, is most likely plesiomorphic at the level of noneudicots. While flowers with such a character combination are rare among extant representatives of the ANA grade (occurring only in Cabombaceae), they are prevalent in magnoliids (e.g. in Piperales, Magnoliales, Laurales) and monocots (e.g. Endress & Doyle, 2009; Endress, 2011; Sauquet *et al*., 2017). Because of their ubiquity and plesiomorphic state, these four floral traits are of limited use when trying to establish the precise phylogenetic placement of *T. monopolyada*.

However, in addition to these plesiomorphic floral traits, most extant Piperales also share a series of less common and likely derived features. As in *T. monopolyada*, the perianth often consists of a single whorl of perianth organs (tepals) with distinctly valvate aestivation in Aristolochiaceae and Hydnoraceae (Soltis *et al*., 2018). However, in contrast to *T. monopolyada*, the tepals of the latter two families are proximally united, forming a perianth tube (Meijer, 1993; Simpson, 2019). In other extant Piperales, the perianth either consists of three free tepals with imbricate aestivation (Lactoridaceae; Kubitzki, 1993), is differentiated into two trimerous whorls (in the genus *Saruma* of Asaraceae; note that in most *Asarum*, the second whorl of tepals, if present at all, is vestigial and that most species in the latter genus have only one whorl of perianth organs; Huber, 1993; Kelly, 2001), or is entirely absent (Piperaceae and Saururaceae; Jaramillo *et al*., 2004). Other noneudicot lineages with a trimerous perianth such as Cabombaceae, monocots, and many other magnoliids are usually characterized by two or more whorls of perianth organs (sometimes differentiated into sepals and petals) with imbricate aestivation patterns (Kadereit, 1993; Loconte, 1993; Williamson & Schneider, 1993; Wu & Kubitzki, 1993a,b; Endress, 2011).

Another distinctive floral trait that is present in *T. monopolyada* is the congenital fusion of stamens and carpels (see Fig. 7b,i–k). This character is rare in angiosperms, but occurs frequently among extant perianth‐bearing Piperales, such as Aristolochiaceae, Lactoridaceae, and Asaraceae (Hou, 1981; Liang & Tucker, 1989, 1995; Endress, 1994, b; González & Stevenson, 2000; Peréz‐Mesa *et al*., 2020). Similar to that found in the flowers of *T. monopolyada*, the stamens are fused to the basal part of the ovary in *Lactoris* (see fig. 3D in Endress, 1994a) as well as in some representatives of *Asarum*, *Saruma*, and *Hydnora* (Endress, 1994a). In the genus *Aristolochia*, with its fully inferior ovary, the stamens are fused with the upper parts of the carpels (González & Stevenson, 2000). The presence of this rare trait in the fossil flower strongly supports a close relationship of *T. monopolyada* with the perianth‐bearing Piperales. Moreover, except for *Lactoris*, all Piperales with a partial androecium–gynoecium fusion possess an inferior or a partially inferior ovary (Huber, 1993; Pabón‐Mora & González, 2024), a trait also observed in *T. monopolyada* where the ovary is inferior to about one third of its length. Outside Piperales, the congenital union of stamens and carpels is also present in Orchidaceae and few other monocots (González & Stevenson, 2000; Rudall & Bateman, 2002). However, the phylogenetic position of *T. monopolyada* within monocots, and in particular within orchids, is unlikely based on clear differences in floral organization (e.g. double perianth in monocots, monosymmetry and reduction in stamens number and a fully inferior ovary in orchids; Endress, 2011) and is also not supported by our phylogenetic analyses (Dataset S1, Fig. S3).

The only monocot lineage that received moderate support as a potential close relative of *T. monopolyada* in one of our analyses (using dataset of Doyle & Endress, 2024) is the genus *Acorus* (Dataset S1, Fig. S3c). *Acorus* is the only genus of Acoraceae and has been identified as the sister lineage of all other monocots (e.g. Zuntini *et al*., 2024). Earlier on, however, *Acorus* was often referred to ‘paleoherbs’, a group of predominantly herbaceous magnoliids and monocots, now recognized as polyphyletic. Next to *Acorus*, the paleoherbs included, among other taxa, also most families now included in Piperales (Tucker & Douglas, 1996; Igersheim & Endress, 1998; Buzgo & Endress, 2000). With *T. monopolyada* as well as with many extant Piperales and with other monocots, *Acorus* shares its trimerous basic floral organization and a series of other floral characters (e.g. whorled perianth phyllotaxis, a whorled androecium phyllotaxis, more than two ovules per carpel; Endress & Doyle, 2009), which most likely are plesiomorphic for mesangiosperms (Sauquet *et al*., 2017). However, an important synapomorphy of the monocot clade (including *Acorus*) is that their flowers are pentacylic with two perianth whorls, two stamen whorls, and one carpel whorl (Remizowa *et al*., 2010). The floral organization of *T. monopolyada*, with its single perianth whorl, three stamen whorls, and one carpel whorl, lacks this monocot synapomorphy. And when comparing the flower of *T. monopolyada* directly with those of *Acorus*, several more differences stand out, including for instance that the flowers of *Acorus* are monosymmetric, the anthers are dorsifixed and introrse, pollen is dispersed as monads and is monosulcate, the ovary is fully superior and contains pendant ovules (Grayum, 1992; Buzgo & Endress, 2000). A closer relationship of *T. monopolyada* with *Acorus* and other monocots is therefore unlikely.

A floral trait that is particularly common in magnoliids and is also present in the antetepalous stamens of *T. monopolyada* is extrorse anther dehiscence, that is, pollen release toward the floral periphery. Among extant angiosperms, the ANA grade is mostly characterized by introrse anther dehiscence (*Cabomba* is extrorse; Doyle & Endress, 2010), which has also been reconstructed as ancestral for the angiosperms as a whole (Endress & Doyle, 2009; Sauquet *et al*., 2017). Along the stem lineage leading to magnoliids, however, anther dehiscence has likely shifted to extrorse (Sauquet *et al*., 2017), which characterizes many magnoliids including Canellales, Magnoliales, and the perianth‐bearing Piperales (Endress & Hufford, 1989; Endress, 1994a). Extrorse anther dehiscence is particularly strongly developed in *Aristolochia*, *Prosopanche*, and Lactoridaceae (Endress, 1994b; González & Stevenson, 2000), whereas latrorse anther dehiscence is typically found in Piperaceae and Saururaceae (Endress, 1994b), as observed in the alternitepalous stamens of *T. monopolyada*.

In summary, our phylogenetic analyses as well as the presence of a series of special floral traits in *T. monopolyada* clearly support a relationship with the magnoliid order Piperales and in particular with the perianth‐bearing subclade of the order (Aristolochiaceae, Asaraceae, and Lactoridaceae). However, as the combination of floral traits found in the fossil flower is not present in the perianth‐bearing clade and as our phylogenetic analyses also find some statistical support for a relationship with other piperalean lineages (Fig. 9c,d), we decided to take a conservative approach and to refer *T. monopolyada* to the order Piperales but not to any specific family therein.

### Additional rare floral traits of *Trimeriantha monopolyada*

*Trimeriantha monopolyada* exhibits a combination of several rare features not observed in extant Piperales, including the presence of three whorls of stamens, flap‐valvate anther dehiscence, pollen polyads, and an extragynoecial compitum. Flowers with three stamen whorls are rare in angiosperms, as most representatives have either one or two stamen whorls (Endress, 2011). However, the inferred ancestral number of stamen whorls for angiosperms as a whole is more than two (Sauquet *et al*., 2017). This feature is found in most representatives of the ANA grade with a whorled floral phyllotaxis (e.g. Nymphaeales, except *Cabomba*, which has a single stamen whorl) and is retained also in some magnoliids (e.g. most Laurales) and eudicots (e.g. some Ranunculales and Proteales) (Endress & Doyle, 2009). These groups usually exhibit a higher number of stamen whorls or numerous spirally arranged stamens. For example, Lauraceae typically have flowers with three whorls of fertile stamens and an inner fourth whorl of staminodes (Rohwer, 1993). Moreover, the number varies considerably both among and within clades, with notable lability in the ANA grade and the magnoliids, where stamen number varies greatly even between closely related taxa (e.g. 6–12 in Lauraceae, up to 1800 in Monimiaceae; Rohwer, 1993; Endress, 2011). Piperales, on the other hand, are generally characterized by two trimerous stamen whorls, except for (e.g. *Lactoris*(Lactoridaceae), Saururaceae p.p.), which have also been reconstructed as ancestral for the order (Endress & Doyle, 2009). However, stamen number and arrangement are diverse across the order, with Aristolochiaceae, for instance, having five to more than 40 stamens arranged in one to four whorls, commonly with six or 12 stamens per whorl, and often with stamen pairs (Huber, 1993; González & Stevenson, 2000; Lu & Wang, 2009).

Anthers with flap‐valvate dehiscence are rare overall and do not occur among extant Piperales. They are often present in Monimiaceae, Lauraceae, and Hernandiaceae within magnoliids, Berberidaceae (except *Nandina*, early diverging eudicots) (Endress & Hufford, 1989) and one genus of Hamamelidaceae (*Hamamelis*, core eudicots; Hufford & Endress, 1989). Among these families, the majority of Lauraceae have tetrasporangiate anthers with two pollen sacs per theca, as present also in *T. monopolyada*, whereas four genera contain bisporangiate anthers with one pollen sac per theca (Endress & Hufford, 1989; Rohwer, 1994). Although *T. monopolyada* shares certain plesiomorphic magnoliid traits and even specific traits (e.g. flap‐valvate dehisced pollen sacs, inaperturate pollen grains) with Lauraceae, differences in several key traits such as the number of perianth whorls, the number of carpels, and the number of ovules clearly distinguish it from this family, which is characterized by two whorls of perianth organs and a unicarpellate ovary containing a single ovule (Rohwer, 1993, 1994). In addition, inaperturate pollen grains (pollen grains without obvious apertures) have evolved independently in different angiosperm lineages (Furness, 2007) and are particularly common among early diverging angiosperm (e.g. Lu *et al*., 2015) and monocots (Furness & Rudall, 1999). Inaperturate pollen is also present in various magnoliids and occurs also in Piperales, especially in Aristolochiaceae but also in Piperaceae p.p. (Sampson, 2000). In Lactoridaceae, apertures are described as small and ‘poorly defined’ (Sampson, 1995; Sampson, 2000).

Flap‐valvate anther dehiscence is relatively rare among extant angiosperms but has been documented in numerous fossil stamens from the Cretaceous, primarily associated with trimerous fossil flowers exhibiting affinities to the Laurales, especially Lauraceae (Drinnan *et al*., 1990; Eklund & Kvaček, 1998; Herendeen *et al*., 1999; Eklund, 2000; Takahashi *et al*., 2001; Frumin *et al*., 2004; von Balthazar *et al*., 2007, 2011; Viehofen *et al*., 2008; Friis *et al*., 2011b). Ancestral state reconstruction shows that early angiosperm stamens likely dehisced via longitudinal slits (Endress, 2009; Sauquet *et al*., 2017). Flap‐valvate dehiscence arose scattered in distantly related lineages in the Early Cretaceous, followed by its increased abundance in the Late Cretaceous (Friis *et al*., 2011b), and may present an early evolutionary specialization that has evolved convergently across the angiosperm phylogeny (Endress, 2009; Sauquet *et al*., 2017).

Another striking feature in *T. monopolyada* is that each pollen sac produces only a single pollen polyad, a type of pollen aggregation found in angiosperms. Different types of pollen aggregates that function as pollen dispersal units (e.g. polyads, pollinia) occur in *c*. 15% of the angiosperm families (Kenrick & Knox, 1982), but pollen polyads – defined as dispersal units of more than four regularly arranged and permanently united pollen grains (Halbritter *et al*., 2018) – are presently documented in only a small number of families, including Annonaceae, Orchidaceae, Celastraceae (including Hippocrateaceae), Fabaceae, Apocynaceae, and Gentianaceae (Walker & Doyle, 1975; Takahashi, 1986; Wyatt *et al*., 2000; Harder & Johnson, 2008; Godoy *et al*., 2012). In the majority of species with pollen polyads in these families, the grain number is 16; only a few species (in Fabaceae) have eight or 32 grains per polyad (Kenrick & Knox, 1982). Pollen tetrads are another form of pollen aggregation that occurs much more frequently than polyads and is also present in *Lactoris* (Lactoridaceae) within the order Piperales (Carlquist, 1964). An interesting trait that *Lactoris* shares with *T. monopolyada* is that the tetrads are calymmate (Sampson, 1995; Stuessy *et al*., 1998; Gamerro & Barreda, 2008), that is, there is a continuous ektexine layer enveloping the entire tetrad. In addition, as in *T. monopolyada*, the individual pollen grains are only loosely packed in the tetrads of *Lactoris* (Sampson, 1995). Walker & Doyle (1975) suggested that polyads have arisen from tetrads, as in every family that possesses polyads, some taxa still retain tetrads, and some polyads consist of individually discernible tetrads. As pollen aggregations are rare but found in distantly related plant groups, they generally do not appear to be indicative of systematic affinities (Carlquist, 1964). However, the combination of calymmate pollen aggregates with individual pollen grains only loosely cohering, as present in *T. monopolyada* and *Lactoris*, is apparently not known from any other angiosperm lineages (Sampson, 1995) and may further support a placement of our fossil flower in Piperales. To our knowledge, the presence of a single pollen polyad per pollen sac, as it is present in *T. monopolyada*, is unique both among extant and other known fossil angiosperms (see also below).

*Trimeriantha monopolyada* possesses a gynoecium with a syncarpous ovary but free styles and stigmas. The latter are arranged closely to each other in the center of the flower and, in specimens that were preserved in an anthetic stage, are covered by the remnants of a common drop of secretion (see Fig. 6c). As the stigmas are entirely covered by secretion, pollen could not be deposited directly on the stigmas but only on the drop of secretion. After pollen germination, pollen tubes had to first grow through the secretion to reach the underlying stigmas. Such a connection of stigmas by secretion functions as an extragynoecial compitum (Endress, 1982, 2011). Extragynoecial compita are generally rare but are found in most families within the ANA grade (all except Trimeniaceae and Cabombaceae), as well as in several families of the orders Laurales, Magnoliales, and Ranunculales (Endress & Igersheim, 2000; Endress & Doyle, 2009). Functionally, an extragynoecial compitum allows for centralized pollen tube competition and the even distribution of pollen tubes among carpels, also in the complete absence of syncarpy and an intragynoecial compitum (Endress, 2011). The majority of clades within the ANA grade and magnoliids (Magnoliales‐Laurales clade) are apocarpous or unicarpellate, whereas in the Canellales‐Piperales clade, syncarpy up to the stigma and an intragynoecial compitum are common (Endress, 2011). However, there are also groups in Piperales, in which the carpels – similar to that found for *T. monopolyada* – are only basally united, having distinct styles and stigmas (e.g. in Lactoridaceae, Saururaceae, and *Saruma* (Asaraceae); Igersheim & Endress, 1998). While extragynoecial compita are apparently absent in these extant Piperales, it is still plausible that it had evolved in *T. monopolyada* as both central pollen tube competition and pollen tube distribution are of high adaptive value (Endress, 1982; Armbruster *et al*., 2002).

### Trait function and pollination biology

Floral functional traits directly impact the reproductive success of plants by determining the efficiency of pollination, fertilization, and seed production (Saunders, 2020). In comparison with extant angiosperms, the flowers of *Trimeriantha monopolyada* possess a series of special and rare features that seem particularly interesting from a functional perspective. While we realize that hypothesizing about the potential pollination biology of a long extinct fossil flower must remain speculative, we still think that it is an instructive exercise in the case of *T. monopolyada* with its many distinctive floral features.

The general floral construction of *T. monopolyada* with short, nonexserted stamens and a small stigmatic area indicates that these flowers were most likely animal‐pollinated. This hypothesis is further supported by the presence of anthers with valvate dehiscence and pollen polyads. Both traits are closely linked to animal pollination among extant angiosperms (Hufford & Endress, 1989; Friis *et al*., 1991; Harder & Johnson, 2008). Further, the small flower size, the presence of valvate anthers, and pollen polyads with a rugulate surface sculpture suggest pollination by minute insects such as for instance thrips or small flies (Faegri & Pijl, 1979; Endress, 2008; Guglielmi & Teixeira, 2024). The rewards for small insects typically include nectar and pollen, which serve as food sources (Faegri & Pijl, 1979). While the flowers of *T. monopolyada* do not have a distinct nectary, the stigmatic secretions and the likely secretory papillae on the carpel surfaces may also have functioned as a reward for pollinators, as for instance in some extant Annonaceae (e.g. *Anaxagorea*, *Uvaria*) (Armstrong & Marsh, 1997; Lau *et al*., 2017). Extant Piperales are pollinated by a range of insect groups, but small flies seem to play an important role in most lineages (Endress, 2010; Gottsberger, 2016).

It is also noteworthy that among the more than 200 flower specimens of *T. monopolyada* in our material, there was not a single one with a widely opened perianth (Dataset S1; Figs S1, S2). Even in specimens that we deemed to have been anthetic at the time of their preservation based on the presence of dehisced anthers and/or stigmatic secretion, the tepals were just slightly open, typically leaving a narrow, trilete opening to the floral center (Fig. 1f). This suggests that the flowers of *T. monopolyada* did not open widely at anthesis. Instead, the tepals likely formed a floral chamber, in which pollination took place, reminiscent of the pollination chambers that are present in some extant Piperales (e.g. in Aristolochiaceae, some *Asarum* of Asaraceae; Boyce, 1992; González & Stevenson, 2000) and in other magnoliids (e.g. in Annonaceae; Endress, 1994, b). The narrow entrance to and the small size of this chamber further support the hypothesis that the pollinators of *T. monopolyada* were small insects such as small flies, small beetles, or thrips. From a functional point of view, pollinators likely entered the floral chamber at or close to the tip of the tepals, where the entrance to the pollination chamber is broadest. With the stigma located just below this entrance, they may have deposited pollen that they brought along from a previous visit to another flower directly on the stigma before continuing further down into the chamber.

Pollen polyads are considered to be of selective advantage in reproduction, as the transfer of multiple pollen grains can be achieved with a single pollinator visit (Kress, 1981; Kenrick & Knox, 1982; Guglielmi & Teixeira, 2024). Another hypothesis suggests that pollen aggregations may have evolved as an adaptation to minimize mixed pollen loads in plants with low pollen–ovule ratios, as in Apocynaceae and Fabaceae (Kenrick & Knox, 1982; Wyatt *et al*., 2000). In *T. monopolyada*, each flower has 36 polyads with 32 pollen grains each and likely nine ovules in total, and thus, a high pollen–ovule ratio of 128 : 1. There are sufficient pollen grains within one pollen polyad to fertilize all the ovules within one ovary. Consequently, the presence of pollen polyads in *T. monopolyada* appears to be primarily adapted to enhance the efficiency of pollen transfer and to increase the success rate of ovule fertilization by possibly rare pollinator visits.

The extragynoecial compitum is generally involved in pollen reception (Saunders, 2020). In *T. monopolyada*, the mean diameter of the drop of secretion forming the extragynoecial compitum (36–51 μm in diameter) exceeds that of an individual pollen polyad (23.3–31.5 μm), while the individual stigmas themselves are much smaller. Consequently, the formation of an extragynoecial compitum enlarges the contact area between pollen polyad and the stigma, enhancing adhesiveness and improving pollen reception efficiency compared to an individual stigma. This suggests that the combination of pollen polyads and extragynoecial compitum may be adaptively linked as they together allow for a full seed set following a single pollination event in *T. monopolyada*.

The unusual floral traits of *T. monopolyada* provide insight into potential pollination strategies and functional diversity of early angiosperms, while also representing a novel combination of traits and reflecting innovation in early floral morphology. Floral disparity in angiosperms is thought to have peaked in the Early Cretaceous and then declined toward the Late Cretaceous (López‐Martínez *et al*., 2024). The outlying position of *T. monopolyada* in morphospace, however, indicates that the floral morphological diversity in the Late Cretaceous has been broader than previously understood. Therefore, our findings provide evidence that the high level of floral morphological diversity may have been sustained and continued to expand in the Late Cretaceous, a pattern also reflected in fossil pollen (Lupia, 1999; Jardine *et al*., 2022). The previous reduction in disparity may also result from the scarcity of fossil records and the frequent exclusion of phylogenetically unstable or unassignable specimens.

### Conclusions

Based on detailed morphological investigations of numerous well‐preserved flower specimens from the Late Cretaceous of Southern Bohemia (Czech Republic), we formally describe a new fossil taxon named *Trimeriantha monopolyada*. Phylogenetic analyses support a relationship with the magnoliid order Piperales. *T. monopolyada* represents the first direct evidence of flowers of Piperales in the Cretaceous. The flowers are tiny, entirely trimerous, bisexual, and characterized by a single whorl of tepals, three whorls of stamens, unique tetrasporangiate stamens with flap‐valvate dehiscence, a single pollen polyad per pollen sac, filament bases fused to the carpels, and a syncarpous gynoecium with a partly inferior ovary and free stigmas covered by an extragynoecial compitum. Such a combination of floral features has not been recorded in any extant or any other extinct taxon. Therefore, *T. monopolyada* further adds to the already known extraordinary morphological diversity (disparity) from the Late Cretaceous record of fossil flowers (Friis *et al*., 2011a). As our morphospace analysis shows, the flowers of *T. monopolyada* belong to the morphologically most divergent angiosperm flowers. The discovery of *T. monopolyada* highlights that extinct angiosperms may exhibit distinctive autapomorphic traits and lineage‐specific floral specializations that are no longer present in living representatives. Our study, therefore, adds further support to the notion that even the Late Cretaceous was a time of high rates of morphological change and character exploration that resulted in numerous flower types with novel character combinations, many of which – like that of *T. monopolyada* – were lost again later in the course of evolution.

## Competing interests

None declared.

## Author contributions

JS, MB and XW designed the study. JS, JK, MB and ZH collected the sediment samples. XW, JS and MB sorted and prepared the fossil materials. FG and SU processed the fossil pollen polyads and conducted palynological analyses. AML‐M performed phylogenetic and morphospace analyses. XW conducted data analysis and drafted the manuscript, with JS and MB contributing to data interpretation. JS and MB revised and finalized the manuscript. All co‐authors reviewed and edited the manuscript.

## Disclaimer

The New Phytologist Foundation remains neutral with regard to jurisdictional claims in maps and in any institutional affiliations.

## Supporting information


**Dataset S1** Morphological datasets for *Trimeriantha monopolyada*.
**Dataset S2** Trait scoring and references for traits added to the matrix of López‐Martínez *et al*. (2023) and Doyle & Endress (2024).

**Fig. S1** Additional specimens of *Trimeriantha monopolyada* that are not shown in the main text.
**Fig. S2** Additional specimens of *Trimeriantha monopolyada* that are not shown in the main text.
**Fig. S3** Phylogenetic positions of *Trimeriantha monopolyada* and their associated uncertainty.
**Fig. S4** Position of Piperales in the floral morphospace and *Trimeriantha monopolyada* in the morphospace through time.
**Table S1** List of the 5% of the most eccentric extant and fossil angiosperm species in the morphospace analysis.

**Notes S1** Matrix of eFLOWER dataset based on López‐Martínez *et al*. (2024).

**Notes S2** Matrix based on Doyle & Endress (2024).

**Notes S3** Backbone tree used for the analyses based on López‐Martínez *et al*. (2023).

**Notes S4** ‘DE MagSister’ tree used for the analyses based on Doyle & Endress (2024).

**Video S1** Three‐dimensional reconstruction of *Trimeriantha monopolyada* sp. nov. (NM‐F 6067) from micro‐CT data.
Please note: Wiley is not responsible for the content or functionality of any Supporting Information supplied by the authors. Any queries (other than missing material) should be directed to the *New Phytologist* Central Office.

## Data Availability

Data are available in Datasets S1, S2, Notes S1–, S4. Tomographic scans are made available publicly at https://phaidra.univie.ac.at/o:2343018.
