# Supplementary material for: Trimerous magnoliid flowers with a unique set of floral and pollen traits from the Late Cretaceous of Southern Bohemia (Czech Republic)
Source: New Phytol. 2026 Jun 2;251(4):2266–83. doi: 10.1111/nph.71310 (PMC13373870; doi:10.1111/nph.71310)
Supplement: Supplementary file 2 — Fig. S1 Additional specimens of Trimeriantha monopolyada that are not shown in the main text. Fig. S2 Additional specimens of Trimeriantha monopolyada that are not shown in the main text. Fig. S3 Phylogenetic positions of Trimeriantha monopolyada and their associated uncertainty. Fig. S4 Position of Piperales in the floral morphospace and Trimeriantha monopolyada in the morphospace through time. Table S1 List of the 5% of the most eccentric extant and fossil angiosperm species in the morphospace analysis. [file NPH-251-2266-s003.pdf]

New Phytologist Supporting Information

Article title: Trimerous magnoliid flowers with a unique set of flower and pollen traits from the Late Cretaceous of Southern Bohemia (Czech Republic)

Authors: Xieting Wu, Maria von Balthazar, Friðgeir Grímsson, Silvia Ulrich, Andrea López-Martínez, Zuzana Heřmanová, Jiří Kvaček, Jürg Schönenberger

Article acceptance date: 14 May 2026

Contents of this document:

Dataset S1 Morphological datasets for *Trimeriantha monopolyada*

Figure S1 Additional specimens of *Trimeriantha monopolyada* that are not shown in the main text.

Figure S2 Additional specimens of *Trimeriantha monopolyada* that are not shown in the main text.

Figure S3 Phylogenetic positions of *Trimeriantha monopolyada* and their associated uncertainty.

Figure S4 Position of Piperales in the floral morphospace and *Trimeriantha monopolyada* in the morphospace through time.

Table S1 List of the 5% of the most eccentric extant and fossil angiosperm species in the morphospace analysis

Dataset S1 : Morphological datasets of *Trimeriantha monopolyada*

1. Traits (1-30) and trait states for the angiosperm-wide data set from Lopéz-Martínez et al. (2023), along with three additional traits (31-33, for scoring and references of see Dataset S2) added in this study for use in the phyloscan and morphospace analyses:

The trait states highlighted in grey represent the trait scoring for *Trimeriantha monopolyada*:

1 (L100_B), structural sex of flower: 0 = bisexual; 1 = unisexual.
2 (L102_B), ovary position: 0 = superior; 1 = inferior.
3 (L201_A), perianth presence: 0 = absent; 1 = present.
4 (L201_B), number of perianth parts: 0 = one to five; 1 = six to ten; 2 = more than ten.
5 (L204_A), fusion of perianth: 0 = free (<5%); 1 = fused (>5%).
6 (L207_A), symmetry of perianth: 0 = actinomorphic; 1 = zygomorphic.
7 (L230_A), perianth phyllotaxy: 0 = whorled; 1 = spiral.
8 (L231_A), number of perianth whorls: 0 = one; 1 = two; 2 = more than two.
9 (L232_A), perianth merism: 0 = dimerous; 1 = trimerous; 2 = tetramerous; 3 = pentamerous.
10 (L234_A), perianth differentiation: 0 = undifferentiated; 1 = differentiated.
11 (L301_B), number of fertile stamens: 0 = one to five; 1 = six to ten; 2 = more than ten.
12 (L305_A), filament: 0 = laminar (wide); 1 = typical (narrow).
13 (L306_A), fusion of filaments: 0 = free (< 5%); 1 = fused (>5%).
14 (L308_A), fusion of filaments to inner perianth series: 0 = free (< 5%); 1 = fused (>5%).
15 (L311_A), anther orientation: 0 = introrse; 1 = latrorse; 2 = extrorse.
16 (L312_A), anther attachment: 0 = basifixed; 1 = dorsifixed.
17 (L313_A), anther dehiscence: 0 = longitudinal slit; 1 = H-valvate; 2 = flap-valvate.
18 (L314_A), connective extension: 0 = absent; 1 = present.
19 (L330_A), androecium structural phyllotaxy: 0 = whorled; 1 = spiral.
20 (L331_A), number of androecium structural whorls: 0 = one; 1 = two; 2 = more than two.
21 (L332_A), androecium structural merism: 0 = dimerous; 1 = trimerous; 2 = tetramerous; 3 = pentamerous.
22 (L400_A), gynoecium phyllotaxy, 0 = whorled; 1 = spiral.
23 (L401_B), number of structural carpels: 0 = one; 1 = two; 2 = three; 3 = four to five; 4 = more than five.
24 (L403_A), fusion of ovaries: 0 = free (< 5%); 1 = fused (> 5%).
25 (L404_B), style differentiation: 0 = absent; 1 = present.
26 (L406_A), fusion of styles: 0 = free (< 5%); 1 = fused (>5%).
27 (L411_A), number of ovules per functional carpel: 0 = one; 1 = two; 2 = three or more.
28 (L412_A), placentation: 0 = axile; 1 = parietal; 2 = free-central; 3 = laminar.
29 (L5000_A), number of apertures: 0 = inaperturate; 1 = monoaperturate; 2 = diaperturate; 3 = triaperturate; 4 = polyaperturate.
30 (L5002_A), aperture shape: ?; 0 = elongate; 1 = pore-like; 2 = colporate.
31 New (W1), partial androecium-gynoecium fusion: 0 = absent; 1 = present.
32 New (W2), extragynoecial compitum: 0 = absent; 1 = present.
33 New (W3), pollen polyads: 0 = absent; 1 = present.

2. Traits (1-154) and trait states for the data set focused on non-eudicots from Doyle & Endress (2024), along with one additional trait (155, gynostemium) added in this study for use in the phyloscan analysis. In addition, we added the trait state "polyad" to the already existing trait number 91 "pollen unit". Note the trait number 115 "extragynoecial compitum" already existed in this data set. For the

scoring of the new trait (partial androecium-gynoecium fusion) and trait state (pollen polyad), we did a broad search of the floral morphological and palynological literature for the taxa in this data set but generally used the same references as for the López-Martínez et al. (2023) matrix (see Dataset S2).

The following characters highlighted in grey for our fossil flower were scored:

1-54 (D1-54): ? (details see Doyle & Endress, 2024).
55 (D55), sex of flowers: 0 = bisexual; 1 = unisexual.
56 (D56), unisexual flower distribution: ? unknown (inapplicable when flowers are bisexual).
57 (D57), floral base: 0 = no hypanthium, superior ovary; 1 = hypanthium, superior ovary; 2 = partially or completely inferior ovary.
58 (D58), floral receptacle (female portion): 0 = short; 1 = elongate.
59 (D59), pits in receptacle bearing individual carpels: 0 = absent; 1 = present.
60 (D60), cortical vascular system: ? 0 = absent or perianth only, 1 = androecium, 2 = A plus G. Deroin (1999): no cortical vascular system.
61 (D61), floral apex: 0 = used up after production of carpels; 1 = protruding in mature flower.
62 (D62), perianth: 0 = present; 1 = absent.
63 (D63), perianth phyllotaxis: 0 = spiral; 1 = whorled.
64 (D64), perianth merism: 0 = trimerous; 1 = dimerous, 2 = polymerous (whorls of more than three).
65 (D65), perianth whorls (series): 0 = one; 1 = two, 2 = more than two.
66 (D66), tepal differentiation: 0 = all more or less sepaloid; 1 = outer sepaloid, inner petaloid; 1 = all petaloid.
67 (D67), petals: 0 = absent; 1 = present.
68 (D68), nectaries on inner perianth parts: ?; 0 = absent; 1 = present.
69 (D69), outermost perianth parts: 0 = free; 1 = at least basally fused.
70 (D70), calyptra derived from the last one or two bracts below flower: 0 = absent; 1 = present.
71 (D71), stamen number: 0 = more than one; 1 = one.
72 (D72), androecium phyllotaxis: 0 = spiral; 1 = whorled.
73 (D73), androecium merism: 0 = trimerous; 1 = dimerous; 2 = polymerous (whorls of more than three).
74 (D74), stamen whorls (series; includes inner staminodes): 0 = one; 1 = two; 2 = more than two.
75 (D75), stamen positions: 0 = single; 1 = double.
76 (D76), stamen fusion: 0 = free; 1 = connate.
77 (D77), androecium symmetry: 0 = actinomorphic; 1 = stamens on anterior side of gynoecium.
78 (D78), inner staminodes: 0 = absent; 1 = present.
79 (D79), glandular food bodies on stamens or staminodes: 0 = absent; 1 = present.
80 (D80), stamen base: 0 = short; 1 = long and wide; 2 = long and narrow (relative to anther).
81 (D81), paired basal stamen glands: 0 = absent; 1 = present.
82 (D82), anther apex: 0 = extended; 1 = truncated or smoothly rounded; 2 = peltate.
83 (D83), pollen sacs: 0 = protruding; 1 = embedded.
84 (D84), number of microsporangia: 0 = four; 1 = two.
85 (D85), orientation of anther dehiscence: 0/2 = distinctly introrse/extrorse; 1 = latrorse-sligtly introrse.
86 (D86), mode of anther dehiscence: 0 = longitudinal slit; 1 = H-valvate; 2 = valvate with upward-opening flaps.
87 (D87), connective hypodermis: ?; 0 = unspecialized; 1 = endothecial or sclerenchymatous.
88 (D88), tapetum: ?; 0 = secretory, 1 = amoeboid.
89 (D89), microsporogenesis: ?.
90 (D90), pollen nuclei: ?.

91 (D91), pollen unit: 0 = monads, 1 = tetrads, 2 = polyads.
92 (D92), pollen size: 0 = large (> 50 μm); 1 = medium (20–50 μm); 2 = small (< 20 μm); ordered.
93 (D93), pollen shape: ?; 0 = boat-shaped; 1 = globose; 2 = triangular, angulaperturate.
94 (D94), aperture: 0 = single; 1 = inaperturate; 2 = sulculate; 3 = Garside tricolpate; 4 = tricolpate.
95 (D95), single aperture shape: ?; 0 = elongate, 1 = round.
96 (D96), single aperture branching: ?; 0 = (mainly) unbranched; 1 = three or more sulcus branches.
97 (D97), infratectum: 0 = granular (including "atectate"); 1 = intermediate, 2 = columellar; ordered.
98 (D98), tectum: 0 = continuous or microperforate; 1 = perforate or semitectate, 2 = reduced.
99 (D99), grading of reticulum: ?.
100 (D100), striate muri: ?; 0 = absent; 1 = present.
101 (D101), supratectal spinules: ?; 0 = absent; 1 = present.
102 (D102), prominent spines (easily visible with light microscopy): ?; 0 = absent, 1 = present.
103 (D103), aperture membrane: ?; 0 = smooth, 1 = sculptured.
104 (D104), nexine: 0 = non-foliated foot layer, no endexine; 1 = foot layer and distinctly staining endexine; 2 = foliated FL.
105 (D105), nexine thickness: 0 = absent or traces; 1 = thin; 2 = thick (1/3 or more of total exine).
106 (D106), carpel number: 0 = one; 1 = 2–5 in 1 whorl (series); 2 = > 5 in 1 whorl; 3 = > 1 whorl.
107 (D107), carpel form: ?; 0 = ascidiate; 1 = intermediate, ovule(s) in ascidiate zone; 2 = plicate.
108 (D108), postgenital sealing of carpel: ?; 0 = none; 1 = partial; 2 = complete.
109 (D109), secretion in area of carpel sealing: ?; 0 = absent; 1 = present.
110 (D110), pollen tube transmitting tissue: ?; 0 = not differentiated; 1 = one cell layer; 2 = > 1 cell layer.
111 (D111), style (elongate, constricted carpel apex): 0 = absent (sessile-capitate stigma); 1= present.
112 (D112), stigma: 0 = extended (half or more of style-stigma zone); 1 = restricted.
113 (D113), multicellular stigmatic protuberances or undulations: ?; 0 = absent; 1 = present.
114 (D114), stigmatic papillae: 0 = absent; 1 = present.
115 (D115), extragynoecial compitum: 0 = absent; 1 = present.
116 (D116), carpel fusion: 0 = apocarpous; 1 = parasyncarpous; 2 = eusyncarpous.
117 (D117), intrusive oil cells in carpels: ?; 0 = absent; 1 = present.
118 (D118), long unicellular hairs on and/or between carpels: 0 = absent; 1 = present.
119 (D119), short curved appressed unlignified hairs on carpels: 0 = absent; 1 = present.
120 (D120), nectary on dorsal or lateral sides of carpel or carpellode: ?; 0 = absent; 1 = present.
121 (D121), septal nectaries or comparable basal intercarpellary nectaries: 0 = absent; 1 = present.
122 (D122), number of ovules per carpel: 0 = one; 1 = 2 or varying between 1 and 2; 2 = more than two.
123 (D123), placentation: 0 = ventral; 1 = laminar-diffuse or "dorsal" (on carpel midline).
124 (D124), ovule direction: 0 = pendent; 1 = horizontal; 2 = ascendent.
125-154 (D125-154): ?; (details see Doyle & Endress, 2024).
155 New(W1), partial androecium-gynoecium fusion: 0 = absent; 1 = present.

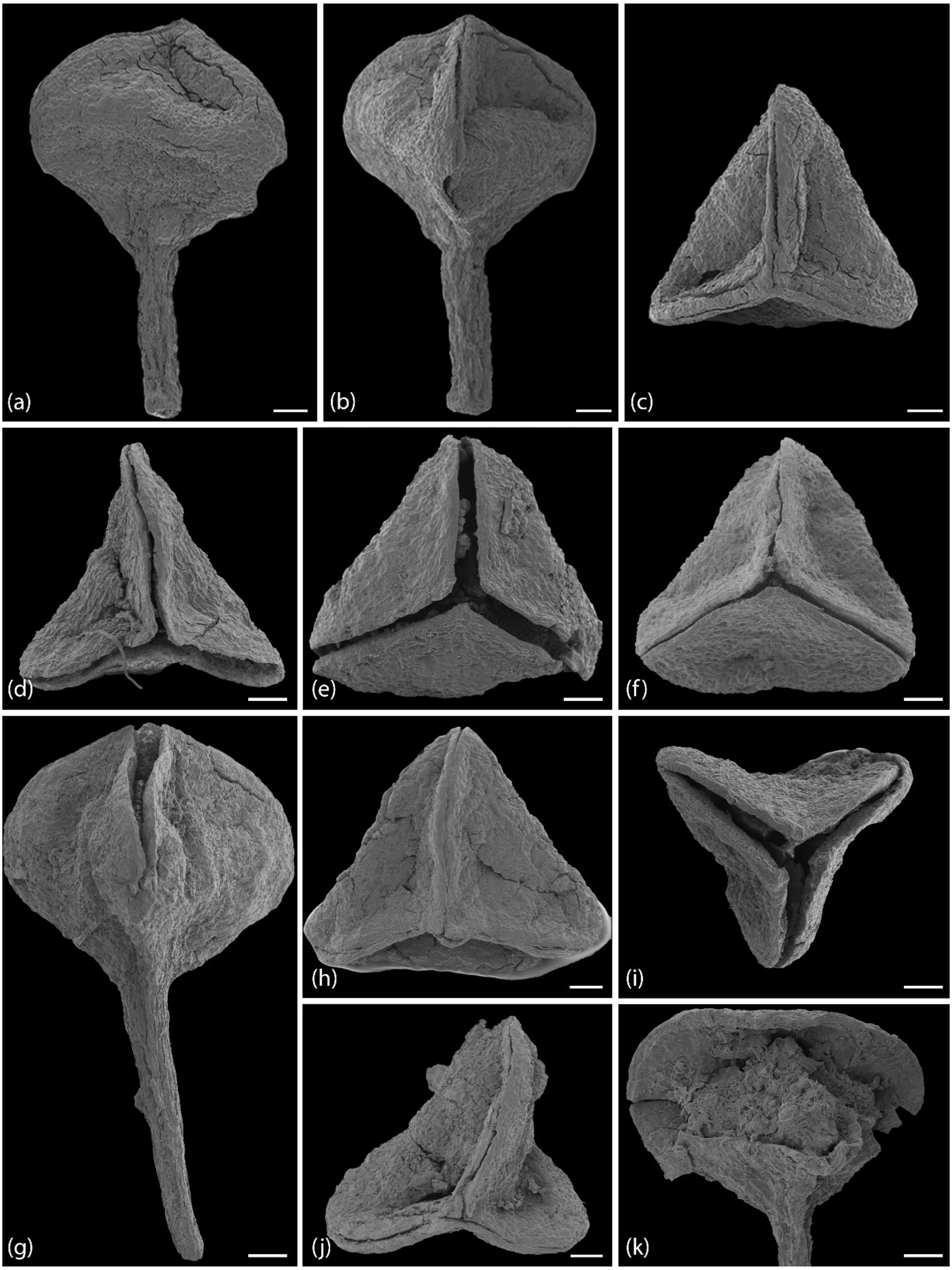

Figure S1. Additional specimens of *Trimeriantha monopolyada* that are not shown in the main text. Different specimens shown in different views. (a-c) NM-F6068. (d) NM-F6088. (e) NM-F6089. (f) NM-F6090. (g) NM-F6091. (h) NM-F6092. (i) NM-F6093. (j) NM-F6094. (k) NM-F6095. Scale bars = 100 μm.

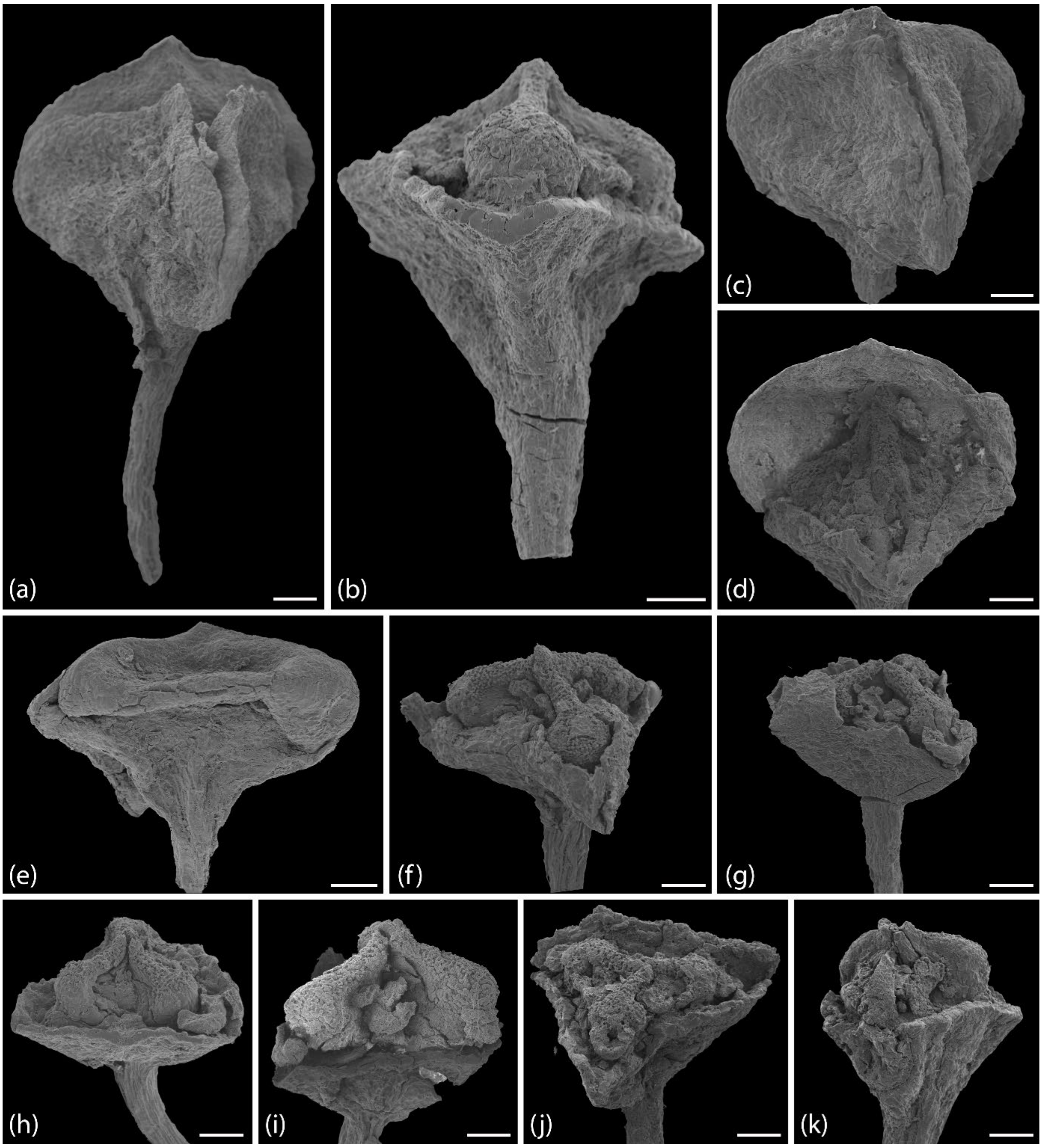

Figure S2. Additional specimens of *Trimeriantha monopolyada* that are not shown in the main text. Different specimens shown in different views. (a) NM-F6096. (b) NM-F6097. (c) NM-F6098. (d) NM-F6099. (e) NM-F6100. (f) NM-F6101. (g) NM-F6102. (h) NM-F6103. (i) NM-F6104. (j) NM-F6105. (k) NM-F6106. Scale bars = 100 μm.

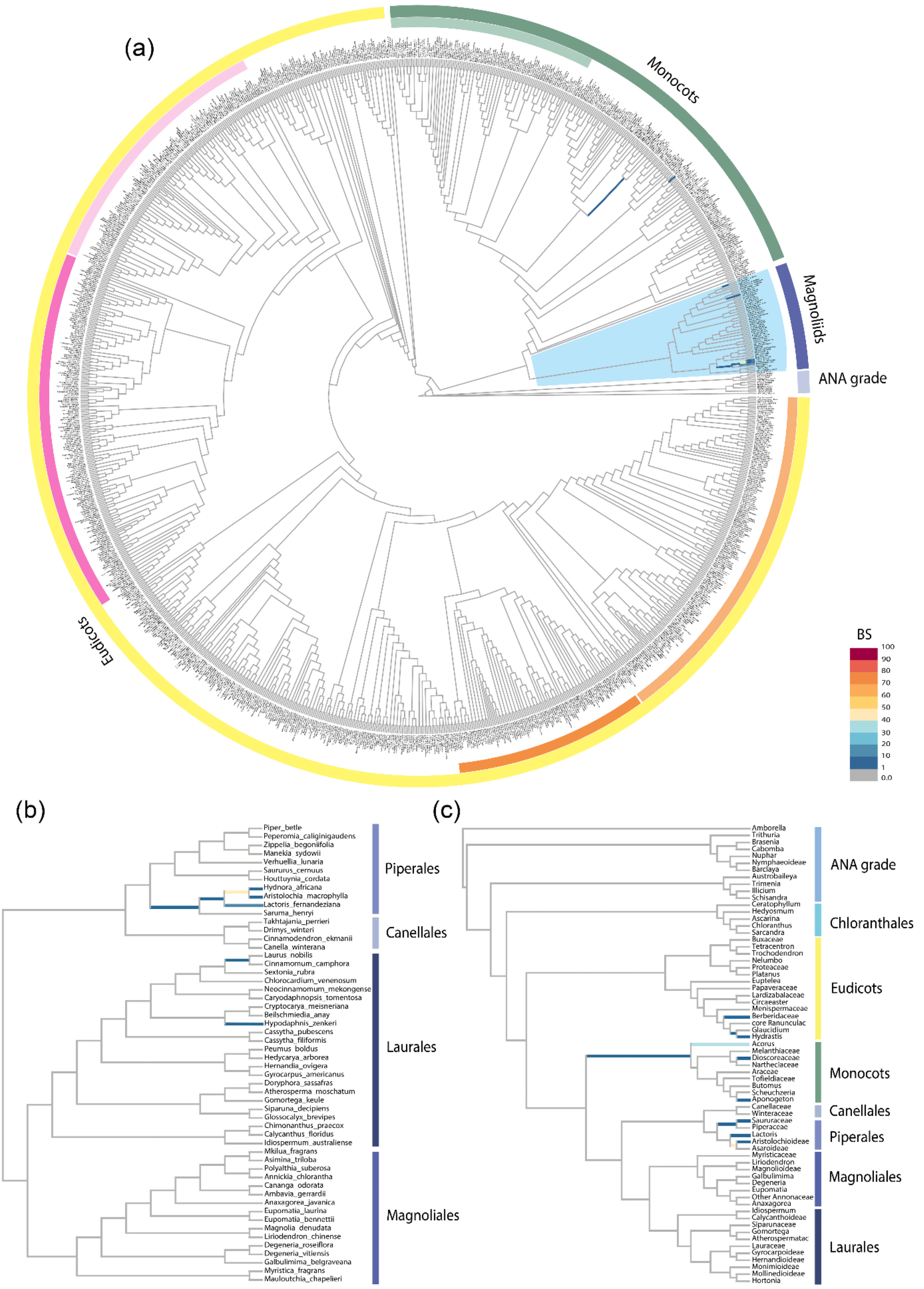

Figure S3. Phylogenetic positions of *Trimeriantha monopolyada* and their associated uncertainty. RoguePlots showing alternative phylogenetic positions of *Trimeriantha monopolyada* and their associated uncertainty. (a) Position of *T. monopolyada* in the entire angiosperm phylogeny based on the maximum likelihood analysis using the combined molecular and morphological matrix of López-

Martínez et al. (2023). (b) Partial RoguePlot from the analysis shown in (a). (c) Partial RoguePlot estimated with the maximum likelihood analysis of the morphological matrix from Doyle and Endress (2024). The branches are colored according to the bootstrap values (BS values in color legend) associated with the attachment of the fossil to the branch.

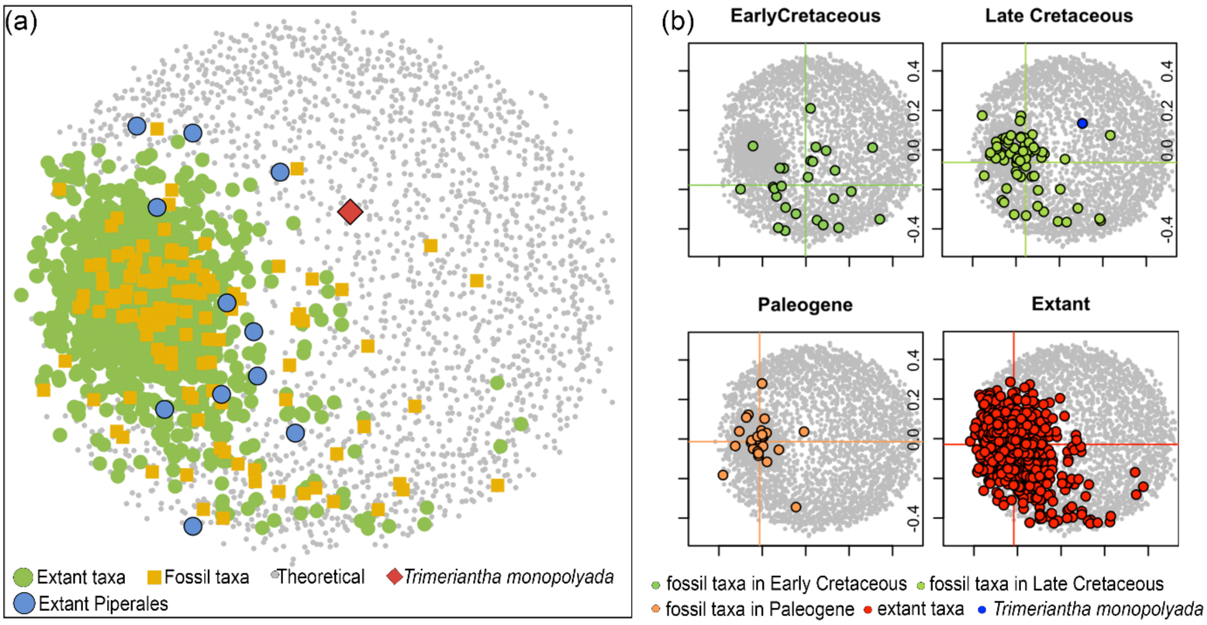

Figure S4. Position of extant Piperales in the floral morphospace and *Trimeriantha monopolyada* in the morphospace through time. (a) Position of extant Piperales (blue dots) and *Trimeriantha monopolyada* (red diamond) in the floral morphospace of angiosperms (based on the study of Lopéz-Martínez et al. 2024); (b) Position of *Trimeriantha monopolyada* (dark blue dot) in the morphospace through time.

Table S1. List of the 5% of the most eccentric species in morphospace analysis, ordered according to their eccentricity (= divergence of each species from the average morphology, see López-Martínez et al. 2024; starting with the most eccentric one; X before a species name denotes fossil taxa). The angiosperm group assigned to the extant taxa, along with the stratigraphic age and the systematic affinity of fossils based on their original description, are indicated.

**Species**	**extant/fossil**	**group**
X Endressinia brasiliana	Early Cretaceous	Magnoliids
Ceratophyllum submersum	extant	Ceratophyllales
Eupomatia laurina	extant	Magnoliids
Eupomatia bennettii	extant	Magnoliids
Galbulimima belgraveana	extant	Magnoliids
Sarcandra chloranthoides	extant	Chloranthales
Ruppia megacarpa	extant	Other Monocots
Idiospermum australiense	extant	Magnoliids
Cercidiphyllum magnificum	extant	Other SuperRosids
Hedycarya arborea	extant	Magnoliids
X Virginianthus calycanthoides	Early Cretaceous	Magnoliids
X Detrusandra mystagoga	Late Cretaceous	Magnoliids
Degeneria vitiensis	extant	Magnoliids
Trithuria cowieana	extant	ANA
X Microvictoria svitkoana	Late Cretaceous	ANA
Trithuria submersa	extant	ANA
X Microaltingia apocarpela	Late Cretaceous	Other SuperRosids
X Platanocarpus brookensis	Early Cretaceous	Other Eudicots
X Archaefructus eoflora	Early Cretaceous	Magnoliids
Cercidiphyllum japonicum	extant	Other SuperRosids
X Schenkeriphyllum glanduliferum	Early Cretaceous	Magnoliids
Cecropia insignis	extant	Fabids
X Archaeanthus linnenbergeri	Early Cretaceous	Magnoliids
Amborella trichopoda	extant	ANA
Piper betle	extant	Magnoliids
Ceratophyllum demersum	extant	Ceratophyllales
Glossocalyx brevipes	extant	Magnoliids
Pandanus tectorius	extant	Other Monocots
Euptelea pleiosperma	extant	Other Eudicots
X Melloniflora virginiensis	Early Cretaceous	Magnoliids
X Lovellea wintonensis	Early Cretaceous	Magnoliids
X Cronquistiflora sayrevillensis	Late Cretaceous	Magnoliids
Peumus boldus	extant	Magnoliids
Typha latifolia	extant	Commelinids
Cyclanthus bipartitus	extant	Other Monocots
X Jerseyanthus calycanthoides	Late Cretaceous	Magnoliids
Austrobaileya scandens	extant	ANA
Calycanthus floridus	extant	Magnoliids

X Cecilanthus polymerus	Late Cretaceous	ANA/Magnoliids
Rafflesia keithii	extant	Fabids
X Platananthus synandrus	Early Cretaceous	Other Eudicots
X Teixeiraea lusitanica	Early Cretaceous	Other Eudicots
Posidonia oceanica	extant	Other Monocots
Schisandra chinensis	extant	ANA
Coptis chinensis	extant	Other Eudicots
Nothofagus cunninghamii	extant	Fabids
Euptelea polyandra	extant	Other Eudicots
X Carpestella lacunata	Early Cretaceous	ANA
Chlorocardium venenosum	extant	Magnoliids
X Trimeriantha monopolyada	**Late Cretaceous**	**Magnoliids**
Gunnera herteri	extant	Gunnerales
Zannichellia palustris	extant	Other Monocots
Nymphaea odorata	extant	ANA
Chimonanthus praecox	extant	Magnoliids
Sararanga sinuosa	extant	Other Monocots
Stylochaeton bogneri	extant	Other Monocots
Casuarina cunninghamiana	extant	Fabids
Xanthosoma sagittifolium	extant	Other Monocots
X Chloranthistemon alatus	Late Cretaceous	Chloranthales
Gymnostoma deplancheanum	extant	Fabids
Mauloutchia chapelieri	extant	Magnoliids
Peperomia caliginigaudens	extant	Magnoliids
Potamogeton berchtoldii	extant	Other Monocots
Aristolochia macrophylla	extant	Magnoliids
Amphibolis griffithii	extant	Other Monocots

LITERATURE CITED

Doyle JA, Endress PK. 2024. Integrating Cretaceous fossils into the phylogeny of living angiosperms: fossil magnoliales and their evolutionary implications. *International Journal of Plant Sciences* **185**: 42–70.

López-Martínez AM, Schönenberger J, von Balthazar M, González-Martínez CA, Ramírez-Barahona S, Sauquet H, Magallón S. 2023. Integrating fossil flowers into the angiosperm phylogeny using molecular and morphological evidence. *Systematic Biology* **72(4)**: 837–855.

López-Martínez AM, Magallón S, von Balthazar M, Schönenberger J, Sauquet H, Chartier M. 2024. Angiosperm reached their highest morphological diversity early in their evolutionary history. *New Phytologist* **241**: 1348–1360.
